# Model-Based Causal Discovery for Zero-Inflated Count Data

**Published:** 2023

**Authors:** Junsouk Choi, Yang Ni

**Affiliations:** Department of Statistics, Texas A&M University, College Station, TX 98195-4322, USA; Department of Statistics, Texas A&M University, College Station, TX 94720-1776, USA

**Keywords:** Bayesian network, Causal identifiability, Directed acyclic graph, Observational zero-inflated count data, Single-cell RNA-sequencing

## Abstract

Zero-inflated count data arise in a wide range of scientific areas such as social science, biology, and genomics. Very few causal discovery approaches can adequately account for excessive zeros as well as various features of multivariate count data such as overdispersion. In this paper, we propose a new zero-inflated generalized hypergeometric directed acyclic graph (ZiG-DAG) model for inference of causal structure from purely observational zero-inflated count data. The proposed ZiG-DAGs exploit a broad family of generalized hypergeometric probability distributions and are useful for modeling various types of zero-inflated count data with great flexibility. In addition, ZiG-DAGs allow for both linear and nonlinear causal relationships. We prove that the causal structure is identifiable for the proposed ZiG-DAGs via a general proof technique for count data, which is applicable beyond the proposed model for investigating causal identifiability. Score-based algorithms are developed for causal structure learning. Extensive synthetic experiments as well as a real dataset with known ground truth demonstrate the superior performance of the proposed method against state-of-the-art alternative methods in discovering causal structure from observational zero-inflated count data. An application of reverse-engineering a gene regulatory network from a single-cell RNA-sequencing dataset illustrates the utility of ZiG-DAGs in practice.

## 1. Introduction

Discovering causal structure of an unknown system is an important task in practically all areas of science. Knowing the causal structure is not only useful for predicting a system’s behavior under external interventions, but also has implications for machine learning tasks such as covariate shift and transfer learning (Schölkopf et al., 2012). The most effective and principled way for causal discovery is to conduct controlled experiments. However, it is often expensive, unethical, or even impossible in certain fields such as genomics (Opgen-Rhein and Strimmer, 2007) and social sciences (Bollen, 1989). Hence, causal discovery approaches that can infer the unknown causal structures from purely observational data are often desired.

This paper considers causal discovery for purely observational zero-inflated count data. Observational zero-inflated count data are common across multiple disciplines, for instance, educational psychology (Fox, 2013), genomics (Kang et al., 2011), ecology (Barry and Welsh, 2002), behavior studies (Hua et al., 2014), and economics (Staub and Winkelmann, 2013). A specific application, by which we are motivated, is to reverse-engineer gene regulatory networks from single-cell RNA-sequencing (scRNA-seq) data. The scRNA-seq technology measures the abundance of mRNA within single cells, resulting in count data with excessive zeros because of technological limits in sequencing the low amounts of mRNA in individual cells. For causal structure learning from observational zero-inflated count data, we work under the framework of causal Bayesian networks (BNs), which have been widely used for representing causal relationships among variables via directed acylic graphs (DAGs).

Learning the structure of BNs is not trivial because the size of the space of possible graph structures grows super-exponentially in the number of variables. Furthermore, BNs may not be distinguishable from each other with observational data. Multiple DAGs can encode the same conditional independence assertions and in general, DAGs are identifiable only up to Markov equivalence class (MEC) in which all DAGs encode the same set of conditional independences (Heckerman et al., 1995). Therefore, in the past, many approaches have focused on identifying the MEC rather than individual DAGs (Spirtes et al., 2000; Chickering, 2002; Kalisch and Bühlman, 2007; Castelletti et al., 2018). For example, the well-known PC algorithm infers a set of conditional independencies and recovers a MEC that is compatible with the inferred conditional independencies (Spirtes et al., 2000). The GES algorithm performs greedy search over the space of MECs and obtains the best-scored MEC (Chickering, 2002). However, DAGs within the same MEC may have drastically different causal interpretations.

Since 2006, it has been shown that for some classes of BNs, the exact graph structure, not just the MEC, may be identifiable from observational data alone. For continuous variables, BNs are often represented by sparse additive noise models. Under this formulation, the underlying DAG is identifiable if the functional form of the additive noise model is linear with non-Gaussian noises (Shimizu et al., 2006; Wang and Drton, 2020) and if the functional form is nonlinear with mild regularity assumptions on the function-noise pair (Hoyer et al., 2008; Peters et al., 2011, 2014). Peters and Bühlmann (2014); Chen et al. (2019) have also shown that unique identification of DAG structure is possible under linear additive noise models with Gaussian noises having equal variances.

The vast majority of the existing works that establish identifiability theorems for BNs have focused on continuous variables; identifiability issues of BNs for count data are less studied. Park and Raskutti (2015) proposed linear Poisson BNs for observational count data and investigated the overdispersion scores to prove that the unique identification of the underlying DAG is possible. However, the applicability of Poisson BNs may be limited due to the restrictive assumption of Poisson distribution that the variance is equal to the mean. Park and Park (2019) generalized the idea of Poisson BNs to a family of generalized hypergeometric distributions that includes the Poisson distribution, the hyper-Poisson distribution, the negative binomial distribution, and many more. An identifiability theorem for the generalized hypergeometric BNs was established using the moment ratio scores. Although the generalized hypergeometric BNs are a quite general class of count BNs, they tend not to adequately model count data with excessive zeros.

There have been a few recent BNs that are fully identifiable for observational zero-inflated data. Using Hurdle conditional distributions, Yu et al. (2020) proposed fully identifiable BNs for zero-inflated Gaussian data. Recently, we (Choi et al., 2020) developed zero-inflated Poisson BNs for observational zero-inflated count data. We have shown, theoretically and empirically, that the underlying causal DAG can be identified from observational data alone. However, the zero-inflated Poisson BNs have the same limitation as Poisson BNs, that is, Poisson distribution is a restrictive distribution. In particular, the Poisson-based BNs do not adequately account for overdispersion, a common feature of count data. Hence it is desirable to further develop a more general class of count BNs that can account for a broad range of multivariate count data with excessive zeros.

In this paper, we introduce a fairly general class of count BNs for observational zero-inflated count data, termed zero-inflated generalized hypergeometric DAGs (ZiG-DAGs). We extend the zero-inflated Poisson BNs (Choi et al., 2020) to zero-inflated generalized hypergeometric models, which include many common count distributions. Therefore, the proposed ZiG-DAGs are capable of modeling various types of zero-inflated count data, for example, overdispersed zero-inflated count data. In addition, we allow for both linear and nonlinear causal relationships in order to flexibly capture real causality in practice whereas Choi et al. (2020) only considers linear causal relationships. Based on a new general proof technique, we prove that the proposed ZiG-DAG is uniquely identifiable, justifying its use for casual discovery. The general proof technique can be potentially used to check identifiability for other discrete BNs as well. The established identifiability theorems do not require the causal faithfulness assumption (Uhler et al., 2013) typically required by constraint-based algorithms. For the structure learning of ZiG-DAGs, we develop score-based algorithms: exhaustive search for small graphs and greedy search for moderate-to-large graphs. Specifically, we consider two different greedy search algorithms to deal with the local optima problem of greedy search. We empirically demonstrate that the proposed methods compare favorably against state-of-the-art alternatives. We also illustrate the utility of ZiG-DAGs in real-world problems using a scRNA-seq dataset.

The remainder of this paper is organized as follows. We set up necessary notations and definitions for BNs in Section 2.1 and we introduce the proposed ZiG-DAG models for observational zero-inflated count data in Section 2.2. Section 3 establishes identifiability theorems for the proposed ZiG-DAGs. In Section 4, we develop score-based algorithms for causal structure learning of ZiG-DAGs. We demonstrate the utility of our methods through synthetic data in Section 5 and real-world applications in Section 6. Section 7 provides our closing discussion.

## 2. Bayesian Networks for Observational Zero-inflated Count Data

### 2.1 Notation and Background

We start with some basic notations for DAGs and BNs. Let $X=\left\{X_{1},\ldots ,X_{d}\right\}$ denote a set of d random variables. A DAG 𝒢=(V,E) consists of a set of nodes V={1,…,d} corresponding to the variables X and a set of directed edges E⊂V×V representing the causal relationships between the nodes V without cycles. If we have a directed edge (j,k)∈E (or slightly abusing the notation, j→k∈E) for j,k∈V, node j is called a parent of k and node k is called a child of j. We denote the set of parents of node j in 𝒢 by $pa_{𝒢}(j)$ and the set of children of j in 𝒢 by $ch_{𝒢}(j)$. Node k is said to be a descendant of node j if there exists a directed path $j=j_{0}\to j_{1}\to \cdots \to j_{l}=k$ and otherwise is said to be a non-descendant of j. We use $nd_{𝒢}(j)$ to denote the set of non-descendants of j (excluding j itself). A BN ℬ for X is a pair ℬ=(𝒢,p) with the joint distribution p(·) factorizing over 𝒢 as follows:

(1) $$p\left(X_{1},\ldots ,X_{d}\right)=\prod_{j=1}^{d}p\left(X_{j}|X_{pa_{𝒢}(j)}\right),$$

where $X_{pa_{𝒢}(j)}=\left\{X_{k}:k\in pa_{𝒢}(j)\right\}$ and $p\left(X_{j}|X_{pa_{𝒢}(j)}\right)$ is the conditional probability distribution of $X_{j}$ given its parents. We say a joint distribution p(·) is (local) Markov with respect to a DAG 𝒢 if each variable $X_{j}$ is independent of its non-descendants $X_{nd_{𝒢}(j)}=\left\{X_{k}:k\in nd_{𝒢}(j)\right\}$ given its parents $X_{pa_{𝒢}(j)}$. The factorization in (1) is equivalent to the Markov property of p(·) (Verma and Pearl, 1990). In this paper, we make the causal Markov assumption -p(·) is Markov with respect to the causal DAG 𝒢 – so that we can interpret 𝒢 causally; in other words, each node is assumed to be independent of all its non-effects conditional on all its direct causes.

**Figure 1:**
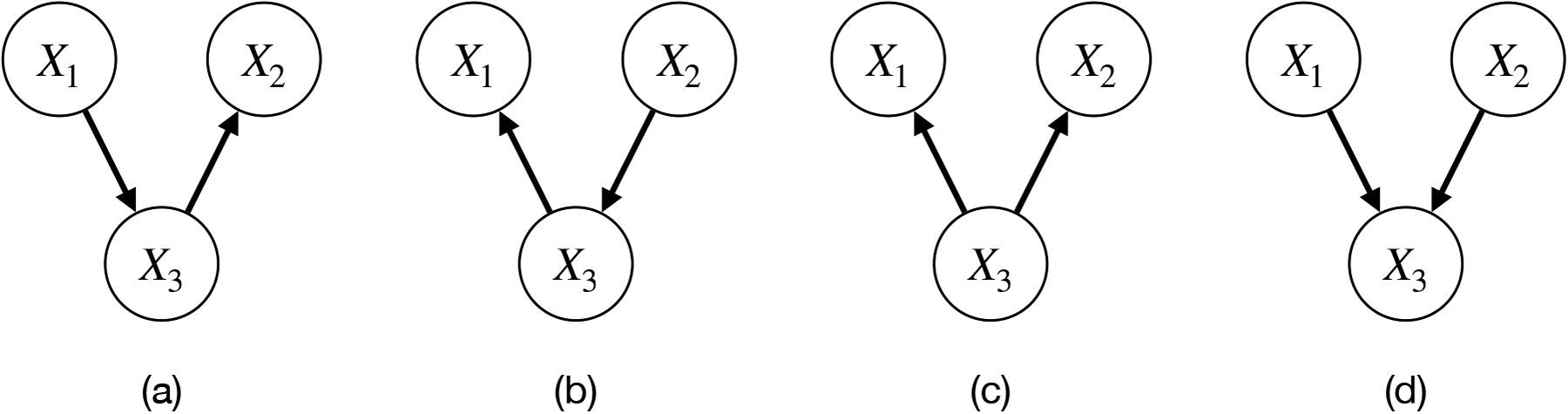
Examples of DAGs with three nodes. DAGs in (a)-(c) are Markov equivalent and form a Markov equivalence class that encodes $X_{1}⫫X_{2}|X_{3}$. DAG in (d) forms another Markov equivalence class that encodes $X_{1}⫫X_{2}$.

In general, the DAG 𝒢 of a BNℬ=(𝒢,p) is not identifiable from the joint distribution p(·). Indeed, the joint distribution p(·) is Markov with respect to many different DAGs including all fully connected DAGs. Therefore, we have many possible BNs with different graph structures for the same joint distribution. To overcome this indeterminacy, one can make additional assumptions and obtain a restricted model for which the graph is identifiable from the joint distribution. A common assumption in the literature for learning BNs is faithfulness. A joint distribution p(·) is faithful with respect to a DAG 𝒢 if the graph 𝒢 encodes all the conditional independence constraints in the joint distribution p(·). If faithfulness is assumed, DAGs are identifiable up to MEC (Spirtes et al., 2000). Two DAGs 𝒢 and ${𝒢}^{\prime }$ are Markov equivalent if the two DAGs encodes the same set of conditional independence constraints and a MEC is defined by a set of DAGs that are Markov equivalent. For example, despite the seemingly different graph structures, the DAGs in Figure 1(a)–(c) forms a MEC, which encodes the only conditional independence $X_{1}⫫X_{2}|X_{3}$, whereas the DAG in Figure 1(d) encodes the marginal independence of $X_{1}$ and $X_{2}$ only and forms another MEC. Since both the Markov property and faithfulness only constrain conditional independencies in the joint distribution, we cannot distinguish DAGs in the same MEC, which impose the same set of conditional independence assertions. For instance, the well-known PC algorithm (Spirtes et al., 2000) and the GES algorithm (Chickering, 2002), under the faithfulness assumption, aim to find the best MEC rather than the best individual DAG.

In many applications of BNs, a specific family of distributions is assumed for the conditional distribution of each node given its parents. For example, we will assume that the conditional probability for each node comes from a zero-inflated count model. Even with such distributional assumptions, the DAG may still be non-identifiable due to the distribution equivalence. Two DAGs 𝒢 and ${𝒢}^{\prime }$ are distribution equivalent if for every BN ℬ=(𝒢,p) there exists a different BN ${ℬ}^{\prime }=\left({𝒢}^{\prime },p^{\prime }\right)$ such that the joint distributions are identical, i.e., $p\left(X_{1},\ldots ,X_{d}\right)=p^{\prime }\left(X_{1},\ldots ,X_{d}\right)$. For example, for Gaussian BNs or multinomial BNs, they are distribution equivalent if and only if they are Markov equivalent. Hence, we can identify only the MEC from the joint distribution for Gaussian and multinomial BNs. Hence, not surprisingly, if we assume that data are generated from one of the three DAGs in Figure 1 (a)–(c), the best answer that we can achieve using Gaussian BNs or multinomial BNs is that one of them is the true model. This is unsatisfactory for many applications and it has been recently shown that there exist certain cases where we can overcome the issue of distribution equivalence and the graph structure is fully identifiable. The existing works often represent continuous BNs as sparse additive noise models and under this framework, the underlying DAG is identifiable if the functional form of the additive noise model is linear and the noises are non-Gaussian (Shimizu et al., 2006), if nonlinear functions are considered with very mild additional conditions (Hoyer et al., 2008; Peters et al., 2011), or if the functions are linear and the noises are Gaussian with equal variance (Peters and Bühlmann, 2014). However, most existing approaches focus on BNs for continuous data, and the identifiability of BNs for count data are much less studied (Park and Raskutti, 2015; Park and Park, 2019; Choi et al., 2020).

### 2.2 Zero-Inflated Generalized Hypergeometric Directed Acyclic Graphs

We consider a broad family of discrete distributions for count data. Kemp (1968a, b) defines a family of generalized hypergeometric probability distributions (GHPDs), which includes a lot of common probability distributions for count data and has many useful properties such as recurrence relationships for both their probabilities and their factorial moments. Let $\left(a{)}_{k}=a(a+1)\cdots (a+k-1\right)$ denote the ascending (rising) factorial with $(a{)}_{0}=1$. The generalized hypergeometric function is then defined as

Fpqa1,…,ap;b1,…,bq;λ=∑i≥0a1i⋯apiλib1i⋯bqii!.

Note that $a_{1},\ldots ,a_{p}$ are exchangeable and so are $b_{1},\ldots ,b_{q}$. A distribution is said to be a GHPD if its probability generating function can be written in the following form:

(2) G(s;a,b,λ)=Fpqa1,…,ap;b1,…,bq;λsFpqa1,…,ap;b1,…,bq;λ,

where $a=\left(a_{1},\ldots ,a_{p}\right)$ and $b=\left(b_{1},\ldots ,b_{q}\right)$. A large number of discrete distributions for count data belong to the class of GHPDs, for example, binomial, Poisson, negative binomial, hypergeometric, beta-binomial, and beta-negative binomial. Table 1 provides some examples of GHPDs with their probability generating functions (see also Kemp 1968a; Dacey 1972; Johnson et al. 2005).

We define, by using the GHPDs, ZiG-DAGs for observational zero-inflated count data. In order to explicitly account for excessive zeros in count data, we adopt the zero-inflated model. We say a BN ℬ=(𝒢,p) for random counts X is a ZiG-DAG if for each node j∈V, the conditional distribution $p\left(X_{j}|X_{pa_{𝒢}(j)}\right)$ of the factorization (1) has a probability generating function of the following form,

(3) $$G_{j}\left(s;X_{pa_{𝒢}(j)}\right)=\pi_{j}\left(X_{pa_{𝒢}(j)}\right)+\left(1-\pi_{j}\left(X_{pa_{𝒢}(j)}\right)\right)\;G\left(s;a_{j},b_{j},\lambda_{j}\left(X_{pa_{𝒢}(j)}\right)\right),$$

where $G\left(s;a_{j},b_{j},\lambda_{j}\right)$ is a GHPD probability generating function defined by (2) with $a_{j}=\left(a_{j1},\ldots ,a_{jp_{j}}\right)$ and $b_{j}=\left(b_{j1},\ldots ,b_{jq_{j}}\right)$. Here, $\pi_{j}(\cdot )$ and $\lambda_{j}(\cdot )$ are functions/mappings from ${𝒳}_{pa_{𝒢}(j)}$ to R, which connect the parents $X_{pa_{𝒢}(j)}$ of node j to its conditional distribution, where ${𝒳}_{pa_{𝒢}(j)}\subset \{0,1,2,\ldots {\}}^{\left|pa_{𝒢}(j)\right|}$. For a ZiG-DAG, the probability mass function of each conditional distribution is given by

$$\text{Pr}\left(X_{j}=x|X_{pa_{𝒢}(j)}\right)\;=\left\{\begin{array}{ll} \pi_{j}\left(X_{pa_{𝒢}(j)}\right)+\left(1-\pi_{j}\left(X_{pa_{𝒢}(j)}\right)\right)\;\text{GHP}\left(0;a_{j},b_{j},\lambda_{j}\left(X_{pa_{𝒢}(j)}\right)\right) & \text{if}\;x=0,\\ \left(1-\pi_{j}\left(X_{pa_{𝒢}(j)}\right)\right)\;\text{GHP}\left(x;a_{j},b_{j},\lambda_{j}\left(X_{pa_{𝒢}(j)}\right)\right) & \text{if}\;x=1,2,\ldots , \end{array}\right.$$

where $\text{GHP}\left(x;a_{j},b_{j},\lambda_{j}\right)$ is the probability mass function of a GHPD of which the probability generating function is given by $G\left(s;a_{j},b_{j},\lambda_{j}\right)$. Particularly, $\pi_{j}(\cdot )\in (0,1)$ is the probability that extra zeros occur in addition to the zeros that arise from the GHPD, and $\lambda_{j}(\cdot )$ is the power parameter of GHPD that is closely related to its moments. For example, in the Poisson probability generating function Gs;aj,bj,λj=F00;;λjs/F00;;λj,λj represents the mean of the Poisson distribution. As another example, in the negative binomial probability generating function Gs;aj,bj,λj=F10k;;λjs/F10k;;λj,λj denotes the probability of “success”, which can be reparametrized in terms of the first and second moments of the negative binomial distribution.

For $\pi_{j}\left(X_{pa_{𝒢}(j)}\right)$ and $\lambda_{j}\left(X_{pa_{𝒢}(j)}\right)$, we consider both linear and nonlinear functional forms. We use the logit function, logit(·), as link function for $\pi_{j}$, where logit(ρ)=log(ρ/(1-ρ)) for 0<ρ<1. Let $h_{j}(\cdot ),j=1,\ldots ,d$, denote any suitable link function for $\lambda_{j}$, which is assumed to be strictly increasing for invertibility. First, we define linear ZiG-DAGs by assuming logit $\left(\pi_{j}\left(X_{pa_{𝒢}(j)}\right)\right)$ and $h_{j}\left(\lambda_{j}\left(X_{pa_{𝒢}(j)}\right)\right)$ vary linearly with the parents $X_{pa_{𝒢}(j)}$ of node j∈V.

**Definition 1 (Linear ZiG-DAGs)** *We say a BN* ℬ=(𝒢,p) *is a linear ZiG-DAG if the joint distribution* p(·) *factorizes with respect to the DAG* 𝒢 *as in* (1) *with each conditional distribution* $p\left(X_{j}|X_{pa_{𝒢}(j)}\right)$ *having a probability generating function* (3)*, where $\pi_{j}\left(X_{pa_{𝒢}(j)}\right)$ and* $\lambda_{j}\left(X_{pa_{𝒢}(j)}\right)$ *are given by*

(4) $$\text{logit}\left(\pi_{j}\left(X_{pa_{𝒢}(j)}\right)\right)=\sum_{k\in pa_{𝒢}(j)}\alpha_{\text{jk}}X_{k}+\delta_{j,}\;h_{j}\left(\lambda_{j}\left(X_{pa_{𝒢}(j)}\right)\right)=\sum_{k\in pa_{𝒢}(j)}\beta_{\text{jk}}X_{k}+\gamma_{j},$$

*with some strictly increasing functions $h_{j}$*.

The zero-inflated Poisson BN in our recent work (Choi et al., 2020) is a special case of the proposed linear ZiG-DAG. Furthermore, in order to allow more flexible causal relationships, we propose nonlinear ZiG-DAGs by adopting the additive model framework. Particularly, for each $X_{j}$, we model $\text{logit}\left(\pi_{j}\left(X_{pa_{𝒢}(j)}\right)\right)$ and $h\left(\lambda_{j}\left(X_{pa_{𝒢}(j)}\right)\right)$ as the sum of nonlinear functions of $X_{k},k\in pa_{𝒢}(j)$.

**Definition 2 (Nonlinear ZiG-DAGs)** *We say a BN* ℬ=(𝒢,p) *is a nonlinear ZiG-DAG if the joint distribution* p *factorizes with respect to the DAG* 𝒢 *as in* (1) *with each conditional distribution* $p\left(X_{j}|X_{pa_{𝒢}(j)}\right)$ *having a probability generating function* (3), *where* $\pi_{j}\left(X_{pa_{𝒢}(j)}\right)$ *and* $\lambda_{j}\left(X_{pa_{𝒢}(j)}\right)$ *are given by*

(5) $$\text{logit}\left(\pi_{j}\left(X_{pa_{𝒢}(j)}\right)\right)=\sum_{k\in pa_{𝒢}(j)}f_{\text{jk}}\left(X_{k}\right)+\mu_{j},\;h_{j}\left(\lambda_{j}\left(X_{pa_{𝒢}(j)}\right)\right)=\sum_{k\in pa_{𝒢}(j)}g_{\text{jk}}\left(X_{k}\right)+\nu_{j},$$

*with some strictly increasing functions $h_{j}$ and nonlinear functions $f_{\text{jk}}$ and* $g_{\text{jk}}$.

Without loss of generality, we assume that $E\left[f_{\text{jk}}\left(X_{k}\right)\right]=E\left[g_{\text{jk}}\left(X_{k}\right)\right]=0,\forall j\in V,k\in pa_{𝒢}(j)$ because they can always otherwise be absorbed into the intercepts $\mu_{j}$ and $\nu_{j}$. If zero-inflated random counts X follow either a linear ZiG-DAG or a nonlinear ZiG-DAG, they satisfy, by definition, the Markov property (conditional independencies) encoded in the underlying DAG. As mentioned earlier, BNs may not be identifiable due to Markov and distribution equivalence. In the next section, we will show that under the proposed ZiG-DAG models, the causal graph structure is identifiable from observational data alone.

## 3. Identifiability Theory

Recently, much effort has been directed to show that some assumptions on the conditional distribution of each node can impose non-independence constraints on the joint distribution so that the DAG of a BN is identifiable (Shimizu et al., 2006; Hoyer et al., 2008; Peters et al., 2014; Peters and Bühlmann, 2014). However, the existing literature mostly addresses the identifiability issue of BNs for continuous data, and there are much fewer identifiability results on BNs for count data. Park and Raskutti (2015); Park and Park (2019) developed BNs by using Poisson and the generalized hypergeometric family and showed that their causal orderings are identifiable. Our recent work (Choi et al., 2020) investigated the identifiability of the zero-inflated Poisson BNs. These three methods are special cases of the proposed ZiG-DAGs; however, none of their proof techniques is applicable in our setting. Therefore, before we state the main identifiability theories for both linear and nonlinear ZiG-DAGs, we provide a general framework to check the identifiability of discrete BNs. We provide a sufficient condition under which two discrete BNs with different DAGs must have different joint distributions. The proofs of the identifiability theorems for the proposed ZiG-DAGs are based on such a sufficient condition. Specifically, Proposition 4 formulates the sufficient condition in terms of probability generating functions for the conditional distribution of each node given its parents. As discrete distributions are often defined by the probability generating function, one can potentially use Proposition 4 to verify the identifiability of other discrete BNs. We first state two assumptions that our identifiability theories require:

**Condition 3** *We assume (i) there exists no unmeasured confounder, and (ii) there is no selection bias*.

No unmeasured confounder (also known as causal sufficiency) and no selection bias are commonly adopted in the literature for causal structure learning (Chickering, 2002; Shimizu et al., 2006; Peters and Bühlmann, 2014; Maathuis et al., 2018). The BN factorization (1) does not hold if either or both assumptions in Condition 3 are violated.

For two discrete BNs ℬ=(𝒢,p) and ${ℬ}^{*}=\left({𝒢}^{*},p^{*}\right)$, we denote $pa(j)=pa_{𝒢}(j)$, $pa^{*}(j)=pa_{{𝒢}^{*}}(j)$, $ch\left(j\right)=ch_{𝒢}\left(j\right)$, ${ch}^{*}(j)={ch}_{{𝒢}^{*}}(j)$, and $nd(j)=nd_{𝒢}(j)$ for the ease of notation. We let $G_{j}\left(s;x_{pa(j)}\right)$ and $G_{j}^{*}\left(s;x_{pa^{*}(j)}\right)$ denote the probability generating functions for the conditional distributions $p\left(x_{j}|x_{pa(j)}\right)$ and $p^{*}\left(x_{j}|x_{pa^{*}(j)}\right)$ of ℬ and ${ℬ}^{*}$, respectively. Note that by definition, $p\left(x_{j}|x_{pa(j)}\right)=G_{j}^{\left(x_{j}\right)}\left(0;x_{pa(j)}\right)$ where $G_{j}^{\left(x_{j}\right)}$ denotes the $x_{j}$-th derivative of $G_{j}$.

**Proposition 4** *Let* ℬ=(𝒢,p) *and* ${ℬ}^{*}=\left({𝒢}^{*},p^{*}\right)$ *be any two discrete BNs, where* 𝒢=(V,E) *and* ${𝒢}^{*}=\left(V,E^{*}\right)$. *Suppose that for every node* j∈V *for which*

(6) $$\frac{G_{j}^{\left(x_{j}+1\right)}\left(0;x_{pa(j)}\right)}{G_{j}^{\left(x_{j}\right)}\left(0;x_{pa(j)}\right)}=\frac{G_{j}^{*\left(x_{j}+1\right)}\left(0;x_{pa^{*}(j)}\right)}{G_{j}^{*\left(x_{j}\right)}\left(0;x_{pa^{*}(j)}\right)}\prod_{k\in ch^{*}(j)\cap \text{nd}(j)}\frac{G_{k}^{*\left(x_{k}\right)}\left(0;x_{pa^{*}(k)\setminus \{j\}},x_{j}+1\right)}{G_{k}^{*\left(x_{k}\right)}\left(0;x_{pa^{*}(k)\setminus \{j\}},x_{j}\right)}$$

*holds for all possible* $x_{1},\ldots ,x_{d}$, *it is also true that* $pa(j)=pa^{*}(j)$ *and* ${ch}^{*}(j)\cap \text{nd}(j)=\emptyset$ *holds. Then, if the joint distributions of* ℬ *and* ${ℬ}^{*}$ *are equivalent, i.e*., $p=p^{*}$, *we have* $E=E^{*}$.

All proofs can be found in the appendices. The main idea behind the proof is to show that if the observational joint distributions p and $p^{*}$ are identical, then the proposition condition (6) necessarily implies that 𝒢 and ${𝒢}^{*}$ have to be identical. Given any topological ordering of the graph 𝒢, we first show that the parent sets, in 𝒢 and ${𝒢}^{*}$, of the last node in the ordering have to be identical if the joint distributions are the same. Then we use mathematical induction to show that this is also true for any node of V and therefore 𝒢 and ${𝒢}^{*}$ have to be identical.

Sometimes, it is also of interest to identify the model parameters. When the graph structure is identifiable, the parameter identifiability simplifies to a question of whether parameters associated with the conditional distribution of each node are identifiable. Since Proposition 4 implies that the graph structure is already identifiable, if a conditional distribution of each node is uniquely determined by the associated parameters, then we necessarily have a one-to-one correspondence between the joint distribution of the BN and the set of all associated parameters, and hence parameters are identifiable.

**Corollary 5** *Let* $\Xi ={\left\{\Xi_{j}\right\}}_{j\in V}$ *and* $\Xi^{*}={\left\{\Xi_{j}^{*}\right\}}_{j\in V}$ *be sets of parameters that are associated with discrete BNs* ℬ *and* ${ℬ}^{*}$, *respectively, where* $\Xi_{j}$ *and* $\Xi_{j}^{*}$ *denote the sets of parameters associated with the conditional distribution of the node* j *only. Suppose that for any* j∈V, *the assumption in Proposition 4 holds and, furthermore*, $\Xi_{j}=\Xi_{j}^{*}$ *whenever* $p\left(x_{j}|x_{pa(j)}\right)=p^{*}\left(x_{j}|x_{pa^{*}(j)}\right)$. *Then, if the joint distributions of* ℬ *and* ${ℬ}^{*}$ *are equivalent, i.e.*, $p=p^{*}$, *we have* $\Xi =\Xi^{*}$.

Using Proposition 4 and Corollary 5, we prove identifiability of the underlying DAG and the associated parameters for both the linear ZiG-DAG and the nonlinear ZiG-DAG in Theorems 6 and 7.

**Theorem 6** *Let* ℬ=(𝒢,p) *be a linear ZiG-DAG. Assume Condition 3 holds. Then, if the variables are not binary, the graph* 𝒢 *is identifiable from the joint distribution* p(X). *For given* $\left(p_{j},q_{j}\right)$ *(which characterizes the generalized hypergeometric function* Fqjpj) *and given* $h_{j}$ *(the link function for* $\lambda_{j}$), *there is a unique set of parameters for the linear ZiG-DAG that induces the observed distribution* p(X).

**Theorem 7** *Let* ℬ=(𝒢,p) *be a nonlinear ZiG-DAG. Assume Condition 3 holds. Then, if the variables are not binary, the graph* 𝒢 *is identifiable from the joint distribution* p(X). *For given ($p_{j},q_{j}$) (which characterizes the generalized hypergeometric function Fqjpj) and given $h_{j}$ (the link function for* $\lambda_{j}$*), there is a unique set of parameters for the nonlinear ZiG-DAG that induces the observed distribution* p(X).

In Theorems 6 and 7, the assumptions that $p_{j},q_{j},h_{j}$ are given, which we make for parameter identifiability, indicates that the conditional distribution of each node in ZiG-DAGs should take a specific GHPD model among the family of GHPDs along with a specific link function. One example of such a combination would be the Poisson distribution with the log link function. Furthermore, the assumption excludes limiting cases of a given GHPD. For instance, if we use the negative binomial distribution for a node, we do not allow it to degenerate to a Poisson distribution, since the negative binomial distribution has $p_{j}=1,q_{j}=0$, while the Poisson distribution has $p_{j}=0,q_{j}=0$. This assumption seems reasonable since we have to decide which GHPD and link function to use in practice. In Sections 5 and 6, for the proposed ZiG-DAG, we consider the Poisson distribution, the hyper-Poisson distribution, and the negative binomial distribution with the log link function. With such choices of GHPD and link function, Theorems 6 and 7 state that both the causal structure and the model parameters for the proposed ZiG-DAGs are fully identifiable from the joint distribution.

Theorems 6 and 7 do not require faithfulness to prove that the exact graph structure is identifiable under the proposed ZiG-DAG models. While continuous BNs such as linear Gaussian BNs may have accidental cancellation of positive and negative causal effects and hence may become unfaithful, the proposed ZiG-DAGs do not allow such cancellation due to inherent asymmetry of count distributions. Faithfulness can be violated in an equilibriummaintaining system such as a biological system (Andersen, 2013) and in datasets with limited sample size (Uhler et al., 2013). In such cases, therefore, causal discovery approaches that require the faithfulness assumption are not favorable. In our specific motivating application of reverse-engineering gene regulatory networks from scRNA-seq data, one should avoid the common practice of “Gaussianizing” raw scRNA-seq data because then one needs to additionally make the faithfulness assumption that may not be suitable in gene regulatory systems; instead, directly working with raw zero-inflated count data with the proposed ZiG-DAG does not suffer from this limitation.

## 4. Algorithms

In this section, we discuss algorithms for learning the causal structures of both the linear ZiG-DAGs and the nonlinear ZiG-DAGs. We will consider score-based approaches, which complement the Bayesian inference procedure developed in our recent work (Choi et al., 2020).

### 4.1 Structure Learning for Linear ZiG-DAGs

Suppose that we are given zero-inflated count data $x=\left\{x^{\left(1\right)},\ldots ,x^{\left(n\right)}\right\}$ that are n independent realizations of X from a linear ZiG-DAG model ℬ=(𝒢,p). For the linear ZiG-DAG, we denote the model parameters by θ={α,β,δ,γ,a,b} with $\alpha ={\left\{\alpha_{\text{jk}}\right\}}_{(j,k)\in E},\beta ={\left\{\beta_{\text{jk}}\right\}}_{(j,k)\in E},\delta ={\left\{\delta_{j}\right\}}_{j\in V},\gamma ={\left\{\gamma_{j}\right\}}_{j\in V},a={\left\{a_{j}\right\}}_{j\in V}$, and $b={\left\{b_{j}\right\}}_{j\in V}$. We let p(·∣θ,𝒢) denote the joint distribution of the linear ZiG-DAG given the model parameters θ and the DAG 𝒢. We score each DAG by the Bayesian information criterion (BIC),

(7) $$\text{BIC}\left(𝒢|x\right)=-2\sum_{i=1}^{n}\text{log}\;p\left(x_{i}|\overset{ˆ}{\theta },𝒢\right)+\left|\theta \right|\;\text{log}\left(n\right),$$

where $\overset{ˆ}{\theta }$ denotes the maximum likelihood estimate of the model parameters and |θ| denotes the number of model parameters. As the individual DAG is identifiable for the proposed ZiG-DAGs, the consistency of the BIC ensures that the true DAG uniquely achieves the minimum BIC with probability converging to 1 as n→∞ (Claeskens et al., 2008). We take two strategies to minimize the BIC given by (7) with respect to the DAG 𝒢: (1) exhaustive search and (2) greedy search.

#### Exhaustive Search

For small graphs where the number of nodes d is small, the BIC can be minimized by computing the scores for all possible DAGs and find the DAG with the lowest score. This approach is exact and is useful for small d (say, d≤4). As the number of nodes d grows, however, this approach becomes computationally infeasible very quickly because the number of DAGs grows super-exponentially in d.

#### Greedy Search

For larger graphs, exhaustive search is infeasible; we will use greedy search instead. Greedy search algorithms in the context of BN learning consider local moves from the current graph and makes the locally optimal choice at each iteration. We consider two strategies, hill climbing (HC) and tabu search (TS) algorithms.

The HC algorithm explores the neighborhood of the current DAG in the space of all possible DAGs. The neighborhood is defined using local moves. At each iteration, the algorithm scores all the DAGs that can be reached from the current graph by an edge addition, deletion, or reversal. The current DAG is then replaced by the DAG that provides the largest improvement, i.e., largest decrease in BIC in our case. We stop the algorithm if the improvement is no longer possible. We summarize the HC procedure in Algorithm 1. Although this algorithm finds a local optimal graph, there is no guarantee that the graph obtained by HC is a global optimum.

In order to avoid being trapped in local optima, the TS algorithm allows s additional local moves (edge addition, deletion, and reversal) when we reach a local optimal graph for which the score cannot be improved. These additional steps explore new territories around the local optimum even if they do not improve the score and may find new direction to arrive at a better structure. Note that the final solution should be the best DAG found anywhere during the search, not the DAG at which the algorithm stops. Furthermore, we keep a list (the tabu list) of all local moves that we have applied within the last t iterations. During the search over the neighborhood of the current DAG, our TS algorithm do not consider local modifications that reverse the local moves in the tabu list. For example, if we add an edge j→k, we cannot delete the edge in the next t steps. This forces the search to explore new directions in the space of DAGs, instead of tweaking with the same parts of the current solution. Our TS algorithm is summarized in Algorithm 2.

**Algorithm 1:** Hill climbing

1:	**Input:** data x, initial DAG ${𝒢}_{0}$.
2:	Compute $\text{BIC}\left({𝒢}_{0}|x\right)$ and set ${\text{BIC}}_{\text{max}}=\text{BIC}\left({𝒢}_{0}|x\right)$.
3:	Set ${𝒢}_{\text{max}}={𝒢}_{0}$.
4:	**repeat**
5:	Initialize Improvement=false.
6:	**for** all DAGs ${𝒢}^{\prime }$ reachable from ${𝒢}_{\text{max}}$ by an edge addition, deletion, or reversal **do**
7:	Compute $\text{BIC}\left({𝒢}^{\prime }|x\right)$.
8:	**if $\text{BIC}\left({𝒢}^{\prime }|x\right)<{\text{BIC}}_{\text{max}}$ then**
9:	Set ${𝒢}_{\text{max}}={𝒢}^{\prime }$ and ${\text{BIC}}_{\text{max}}=\text{BIC}\left({𝒢}^{\prime }|x\right)$.
10:	Set Improvement=true.
11:	**end if**
12:	**end for**
13:	**until Improvment** is false
14:	**Output:** DAG ${𝒢}_{\text{max}}$.

**Algorithm 2:** Tabu search

1:	**Input:** data x, initial DAG ${𝒢}_{0}$, number of additionally allowed steps s, size of the tabu list t.
2:	Compute $\text{BIC}\left({𝒢}_{0}|x\right)$ and set ${\text{BIC}}_{\text{max}}=\text{BIC}\left({𝒢}_{0}|x\right)$.
3:	Set ${𝒢}^{*}={𝒢}_{\text{max}}={𝒢}_{0}$.
4:	Initialize LastImprovement=0.
5:	**while** LastImprovement<s **do**
6:	Initialize ${\text{BIC}}^{*}=\infty$.
7:	**for** all DAGs ${𝒢}^{\prime }$ reachable from ${𝒢}^{*}$ by an edge addition, deletion, or reversal **do**
8:	**if** ${𝒢}^{\prime }$ does not reverse local moves in the tabu list, (i.e., in the last t steps) **then**
9:	Compute $\text{BIC}\left({𝒢}^{\prime }|x\right)$.
10:	**if** $\text{BIC}\left({𝒢}^{\prime }|x\right)<{\text{BIC}}^{*}$ **then**
11:	Set ${𝒢}^{*}={𝒢}^{\prime }$ and ${\text{BIC}}^{*}=\text{BIC}\left({𝒢}^{\prime }|x\right)$.
12:	**end if**
13:	**end if**
14:	**end for**
15:	**if** ${\text{BIC}}^{*}<{\text{BIC}}_{\text{max}}$ **then**
16:	Set ${𝒢}_{\text{max}}={𝒢}^{*}$ and ${\text{BIC}}_{\text{max}}={\text{BIC}}^{*}$.
17:	Set LastImprovement=0.
18:	**else**
19:	Set LastImprovement=LastImprovement+1.
20:	**end if**
21:	**end while**
22:	**Output:** DAG ${𝒢}_{\text{max}}$.

### 4.2 Structure Learning for Nonlinear ZiG-DAGs

While our identifiability theory for nonlinear ZiG-DAGs is general, for structure learning, we need to make specific choice of the nonlinear functions $f_{\text{jk}}$ and $g_{\text{jk}}$. For example, one can expand $f_{\text{jk}}$ and $g_{\text{jk}}$ with the Fourier bases if the functional relationship is expected to be periodic. Similarly, if we expect that the relationship might show a very localized behavior, wavelets can be a good choice. In this paper, we employ spline basis expansion for $f_{\text{jk}}$ and $g_{\text{jk}}$. Splines are popular in semiparametric function estimation because of the ease of their construction, their flexibility and accuracy to approximate a smooth function, and their interpretability through the representation by a compact set of basis functions and coefficients. Particularly, $f_{\text{jk}}$ and $g_{\text{jk}}$ are modeled by cubic B-splines,

$$f_{\text{jk}}(\cdot )=\sum_{l=1}^{M_{f}}\zeta_{\text{jkl}}B_{\text{jkl}}(\cdot )\;\text{and}\;g_{\text{jk}}(\cdot )=\sum_{l=1}^{M_{g}}\eta_{\text{jkl}}C_{\text{jkl}}(\cdot ),$$

where ${\left\{B_{\text{jkl}}(\cdot )\right\}}_{l=1}^{M_{f}}$ and ${\left\{C_{\text{jkl}}(\cdot )\right\}}_{l=1}^{M_{g}}$ are cubic B-spline basis functions with some pre-specified knots. In summary, the nonlinear ZiG-DAG model is parameterized by spline coefficients $\zeta_{\text{jkl}},\eta_{\text{jkl}}$ and the other node-specific model parameters $\mu_{j},\nu_{j},a_{j},b_{j}$. The BIC for each DAG can be evaluated in the same way with (7) and we can use either exhaustive search or greedy search as in Section 4.1 for estimating the underlying graph for nonlinear ZiG-DAGs. The R implementation of the proposed method is available in the R package ZiGDAG (https://github.com/junsoukchoi/ZiGDAG.git).

## 5. Experiments

We empirically evaluate the causal discovery performance of both linear and nonlinear ZiG-DAG models with synthetic data. We compare the proposed method with state-of-the-art BN learning algorithms for count data: the overdispersion scoring (ODS) algorithm for Poisson BNs (Park and Raskutti, 2015) and the moments ratio scoring (MRS) algorithm for generalized hypergeometric BNs (Park and Park, 2019). We also consider the ZiDAG for zero-inflated Gaussian data (Yu et al., 2020) with the log(x+1) transformation of the synthetic count data.

### 5.1 Linear ZiG-DAG

We first consider a linear ZiG-DAG, where the conditional distribution of each node has a probability generating function given by (3) with Gs;aj,bj,λj=F111;ψj,λjs/F111;ψj,λj. That is, the conditional distribution of each node follows a zero-inflated hyper-Poisson, which is a quite flexible distribution as the hyper-Posson distribution allows for both overdispersion and underdisperion in count data. We sample data from the linear ZiG-DAG with different sample sizes n∈{250,500,1000,2000} and different numbers of nodes d∈{10,25,50,100}. For each simulation setting, we set the causal DAG 𝒢 by randomly generating a sparse DAG with d edges. Given the DAG, we generate coefficients ($\alpha_{\text{jk}},\beta_{\text{jk}}$) in (4) from independent uniform distributions: $\alpha_{\text{jk}}\sim U(0.5,2)$ and $\beta_{\text{jk}}\sim U(-2,-0.5)$ for $k\in pa_{𝒢}(j)$ and j∈V. The intercepts $\delta_{j}$ and $\gamma_{j}$ in (4) are chosen uniformly at random from (−1.5, 1) and (1, 1.5), respectively. The additional parameters $\psi_{j}$ for the GHPD (hyper-Poisson distribution) are sampled as $\text{log}\left(\psi_{j}\right)\sim U(-2,2)$. These ranges are chosen so that the resulting observations are not all zeros or do not have extremely large values. Each simulation setting is repeated 50 times, and the simulated datasets have ~ 50% zeros.

For ZiG-DAG, we implement both HC and TS algorithms as introduced in Section 4. Since they are greedy, initial values can affect the outcome. We consider two ways of initialization: first, we start HC (HC0) and TS (TS0) at the empty graph; and second, we initialize HC (HC1) and TS (TS1) with the DAGs obtained by MRS, which are expected to be better than empty graphs. To assess the causal discovery performance of each method, we calculate the true positive rate (TPR), the false discovery rate (FDR), and the Mattews correlation coefficient (MCC) for selection of true directed edges. MCC is a balanced measure of binary classification that takes a value between −1 and 1 with 1 indicating perfect agreement between the true and estimated graphs (i.e., perfect selection), 0 indicating random guess, and −1 indicating total disagreement.

We summarize in Tables 2–3 the operating characteristics of each method for different combinations of the sample size n and the number of nodes d. For every simulation setting, the proposed methods consistently outperform ODS, MRS, and ZiDAG. Specifically, as the sample size increases, our greedy search algorithms find the causal structure more accurately as expected. Our approaches also show satisfactory performance for various graph sizes including moderately large graphs (d=100). We make additional observations for difference between the HC and TS algorithms. When the greedy search algorithms starts at the empty graph, i.e., HC0 and TS0, the performance of TS is better than that of HC. However, if we consider HC1 and TS1 for which we provide more informative initial DAG, there is no statistically significant difference between HC1 and TS1 in most cases. In subsequent simulations, for simplicity, we leave out HC1, TS0 and TS1, and only consider HC0 to learn the proposed ZiG-DAGs from data.

#### Model Misspecification

**Figure 2:**
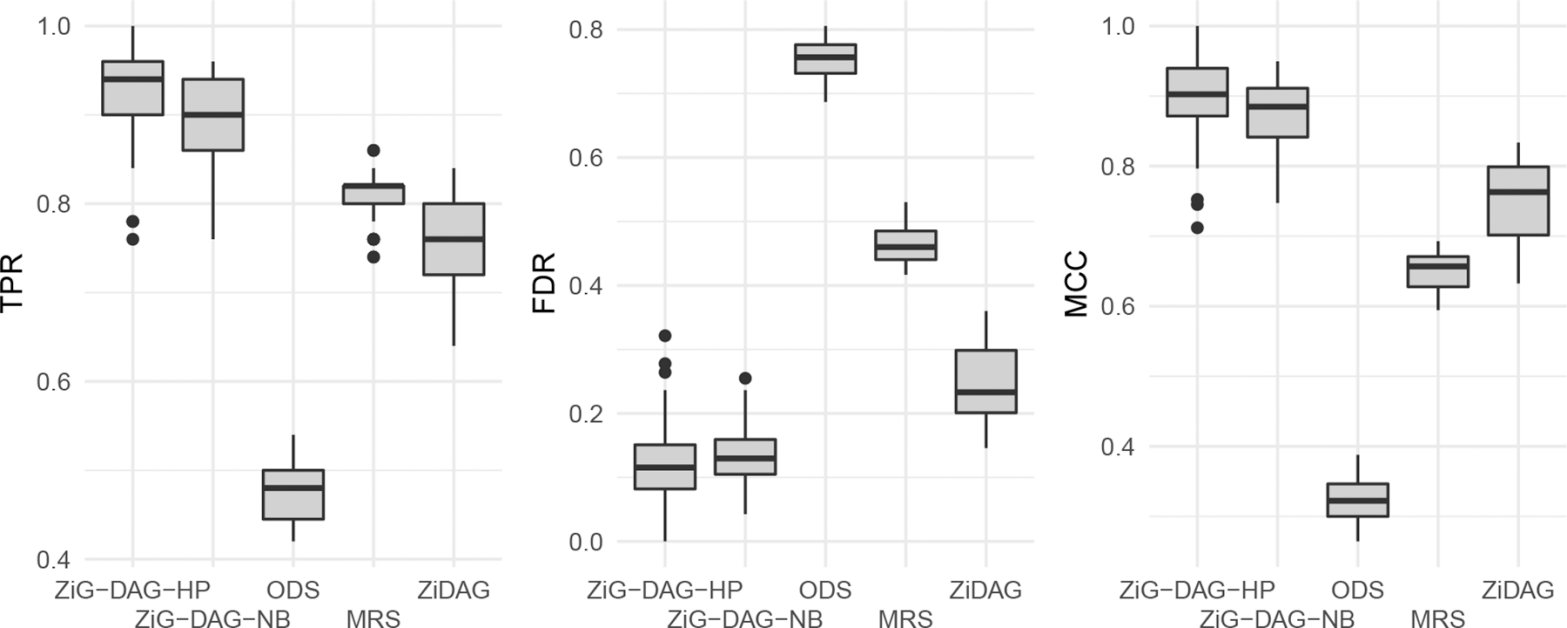
Box plots of operating characteristics for ZiG-DAG-HP, ZiG-DAG-NB, ODS, MRS, and ZiDAG applied to synthetic datasets generated from a linear ZiG-DAG with n=1000 and d=50.

When the conditional distribution of each node in a ZiG-DAG model is misspecified, our identifiability theories do not guarantee that we can find the true DAG. Therefore, an important question is how well our algorithms recovers the true graph when misspecified distributions are used. We investigate this empirically. We choose the simulation scenario with n=1000 and d=50, and apply two different linear ZiG-DAG models. The first one is a linear ZiG-DAG (ZiG-DAG-HP) using the zero-inflated hyper-Poisson distribution as above. The second one is another linear ZiG-DAG (ZiG-DAG-NB) where the conditional distribution of each node is assumed to follow a zero-inflated negative binomial distribution. In ZiG-DAG-NB, every conditional distribution is misspecified, as the true data-generating model is ZiG-DAG-HP. The simulation results are shown in Figure 2. Both ZiG-DAG-HP and ZiG-DAG-NB are better than ODS, MRS, and ZiDAG. Although ZiG-DAG-NB is a misspecified model, its performance is still better than the alternative state-of-the-art approaches. This shows that the proposed ZiG-DAG is useful for learning the true causal structure even if the true conditional distributions are misspecified.

### 5.2 Nonlinear ZiG-DAG

We next assess the performance of the nonlinear ZiG-DAG models. We sample data from a nonlinear ZiG-DAG with n=500 and d=10, where the conditional distribution of each node is again assumed to be a zero-inflated hyper-Poisson. We randomly choose the true nonlinear functions $f_{\text{jk}}$ and $g_{\text{jk}}$ in (5) from three candidates, respectively:

$$f_{1}\left(z\right)=\frac{1}{2}z\left(z-3\right),\;f_{2}\left(z\right)=\text{sin}\left(z\right),\;f_{3}\left(z\right)=\text{exp}\left(\frac{1}{2}z-1\right),$$

and

$$g_{1}\left(z\right)=-\frac{1}{2}{\left(z-\frac{3}{2}\right)}^{2},\;g_{2}\left(z\right)=\text{cos}\left(z\right),\;g_{3}\left(z\right)=-\frac{1}{2}\text{log}\left(z+1\right).$$

The intercepts $\mu_{j},\nu_{j}$ in (5) and the additional parameters $\psi_{j}$ for the hyper-Poisson are generated as in Section 5.1: $\mu_{j}\sim U(-1.5,-1)$, $\nu_{j}\sim U(1,1.5)$, and $\text{log}\left(\psi_{j}\right)\sim U(-2,2)$. For learning the nonlinear ZiG-DAG, we use $M_{f}=M_{g}=4$ spline basis with a knot being placed at the 50% quantile of the data. We also consider the linear ZiG-DAG for comparison. Additionally, since ZiDAG allows for both linear and nonlinear causal relationships, in this simulation study, we use ZiDAG with nonlinear implementation.

We report in Table 4 the simulation results based on 50 repetitions. Overall, the nonlinear ZiG-DAG outperforms the other approaches including the linear ZiG-DAG. Especially, the nonlinear ZiG-DAG results in extremely low FDR compared to the other competitors. Not surprisingly, in this nonlinear simulation setting, ZiDAG gives better results than the linear ZiG-DAG.

### 5.3 Non-zero-inflation

Although the proposed ZiG-DAG models are primarily developed to deal with excessive zeros in count data, they are also applicable and robust to count data generated from non-zero-inflated distributions. We perform additional simulations to support this claim. We generate data from a negative binomial BN, which does not include any zero-inflation components. The parameters for the negative binomial BN are sampled uniformly at random in a similar way to Section 5.1. The resulting data have ~ 26% zeros, which are much less than the zero-inflated case. As in Section 5.1, we consider ZiG-DAG-HP and ZiG-DAG-NB that assume a zero-inflated hyper-Poisson distribution and a zero-inflated negative binomial distribution for the conditional distribution of each node, respectively. Furthermore, we consider two distinctive MRS algorithms that learn DAGs for hyper-Poisson BNs (MRS-HP) and negative binomial BNs (MRS-NB). Since MRS-NB requires an input of the dispersion parameter of the negative binomial distribution, we provide it with the true dispersion parameter value.

The simulation results are shown in Table 5. Even though the data are not zero-inflated, our approaches, ZiG-DAG-HP and ZiG-DAG-NB, generally show better performance than the alternative methods (ODS, MRS-HP, MRS-NB, and ZiDAG). Even though MRS-NB uses the correct distributional model and the true dispersion parameter, it shows worse performance than our methods as well as ZiDAG with respect to FDR and MCC. This might be because the performance of the MRS algorithm highly relies on the choice of external methods for the skeleton estimation. In our experiments, MRS utilizes the R package MXM to estimate the skeleton of DAG, which might provide unreliable skeleton estimates in this simulation setting.

### 5.4 Latent Confounders

**Figure 3:**
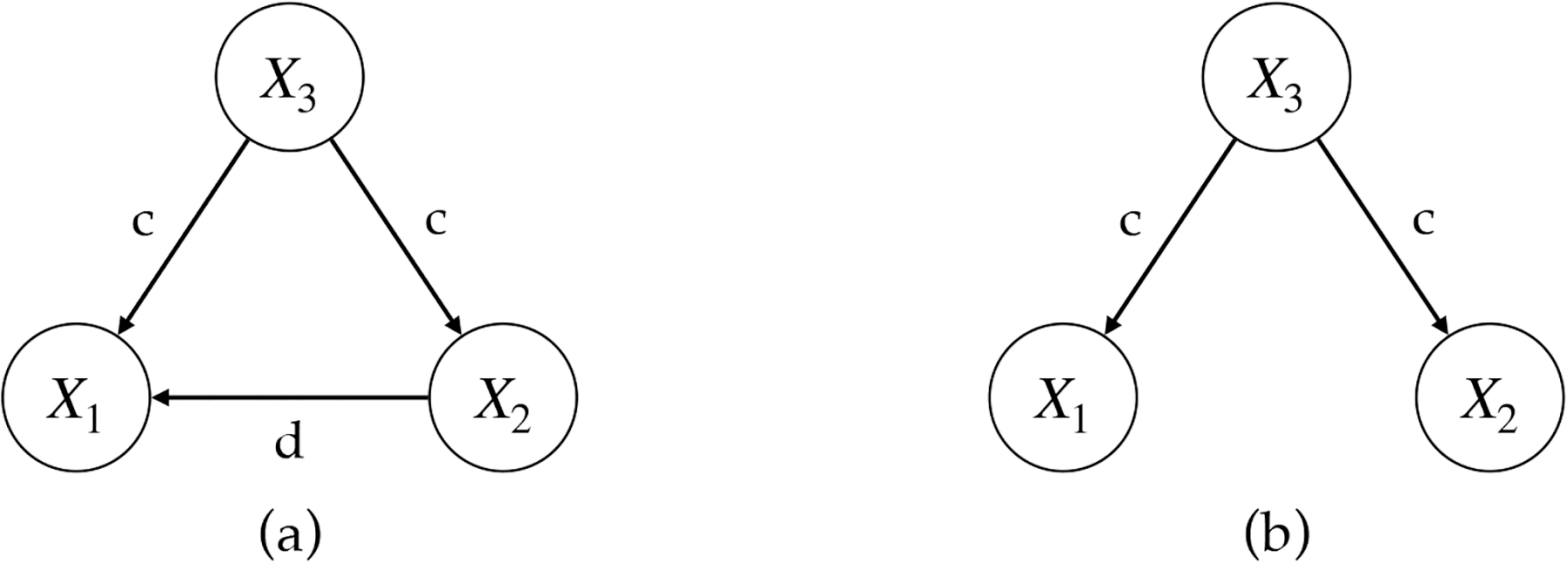
Two different confounding scenarios with a confounder $X_{3}$.

Recall that Theorems 6 and 7 in Section 3 assume causal sufficiency (Condition 3), that is, there exist no latent confounders. Although the causal sufficiency assumption is common in the causal literature, in real applications, it is difficult to check whether an unmeasured latent confounder exists, and there is always a possibility that we do not observe some variables of interest. Therefore, we test how sensitive our method is to the existence of latent confounders. We consider two true causal DAGs in Figure 3 that have three nodes, $X_{1},X_{2},X_{3}$. Given each causal graph, we generate zero-inflated count data from a linear ZiG-DAGs and treat $X_{3}$ as an unmeasured confounder (i.e., hide it from the algorithms).

**Figure 4:**
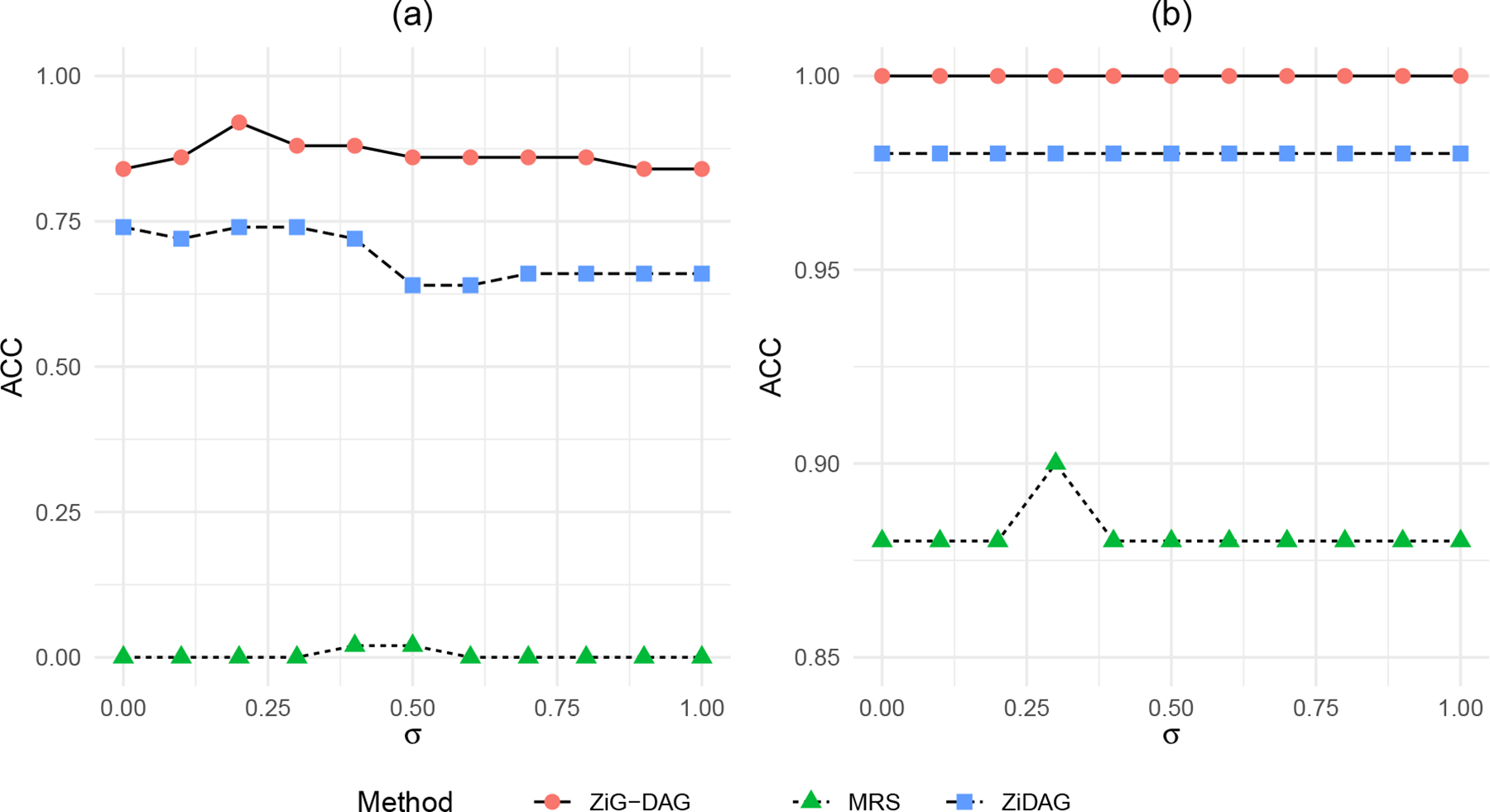
Plots of average ACC of ZiG-DAG, MRS, and ZiDAG against different levels of confounding effect σ under the confounding scenarios of (a) Figure 3(a) and (b) Figure 3(b).

The graph in Figure 3(a) assumes a casual effect of $X_{2}$ on $X_{1}$, which is confounded by $X_{3}$. For the simulation truth corresponding to Figure 3(a), we assume that the conditional distribution of each node is a zero-inflated hyper-Poisson similarly to Section 5.1. We denote $c=\left(\alpha_{13},\beta_{13}\right)=\left(\alpha_{23},\beta_{23}\right)$ and $d=\left(\alpha_{12},\beta_{12}\right)$, and set $\delta_{j}=-1,\gamma_{j}=0$ and $\psi_{j}=5$. We consider different levels of confounding effects c=σ×(-0.8,0.8) where σ∈{0,0.1,0.2,…,1}, while fixing the causal effect d=(0.8,-0.8). For each level of confounding effect, we simulate 50 datasets with sample size n=250. Figure 4(a) plots the average accuracy (ACC) over 50 repeat simulations of ZiG-DAG for identifying the true causal direction $X_{2}\to X_{1}$. We also consider MRS and ZiDAG as benchmarks. Our approach finds the true causal direction quite well across the confounding levels, while ZiDAG becomes worse when the confounding effect is relatively large (σ>0.5). MRS does not work well in this case.

Next, we consider the DAG in Figure 3(b). There is no causal effect between $X_{1}$ and $X_{2}$ whereas the confounding effect by $X_{3}$ is still present. Therefore, we set d=0; otherwise the same simulation truth with Figure 3(a) is used. We consider the same confounding effects with Figure 3(a). Figure 4(b) displays the resulting ACCs of ZiG-DAG, MRS, and ZiG-DAG, again averaged over 50 repeat simulations. In the range of the confounding level being considered, ZiG-DAG does not add any spurious causal relation between $X_{1}$ and $X_{2}$. In summary, the empirical results in Figure 4(a)–(b) indicate that the proposed ZiG-DAG is relatively robust to the presence of hidden confounders.

## 6. Real Data Analyses

We illustrate the utility of the proposed ZiG-DAG by performing two analyses of a scRNA-seq dataset (Li et al., 2017) that consists of 561 cells from 11 primary colorectal cancer (CRC) tumors and matched normal mucosa.

### 6.1 Real-Data Validation with Known Causal Relationships

Using the real scRNA-seq data and known causal relationships in the biological literature, we validate the causal identifiability of ZiG-DAG and compare it to other state-of-the-art alternatives. First, from the TRRUST database (Han et al., 2018), we extract a list of literature-curated pairs of transcription factor and its target. This list establishes a biological ground truth of cause-and-effect relationships with the transcription factors being causes and the targets being effects. We extract from our scRNA-seq data the pairs of genes on the list for which the maximum information coefficient (Reshef et al., 2011), a measure of linear and nonlinear correlations between two variables, is greater than 0.5. This results in 47 pairs for validation.

We apply the proposed ZiG-DAG to each pair of genes. Specifically, we use a nonlinear ZiG-DAG where the conditional distribution of each node is a zero-inflated hyper-Poisson distribution. For comparison, we apply MRS and ZiDAG to the same dataset. We calculate the accuracy of identifying true causal relationships for ZiG-DAG, MRS, and ZiDAG, and the results are 60%, 51%, and 53%, respectively. Out of a total of 47 pairs, the proposed ZiG-DAG correctly identifies 28 causal relationships. This indicates that the proposed method is capable of finding true causal relationships in real data: the p-value for a binomial test is 0.0002 when compared to random guesses. Furthermore, among the three count BNs, ZiG-DAG has the highest accuracy.

### 6.2 Reverse Engineering of Gene Regulatory Network

In this section we aim to reconstruct a gene regulatory network for d=26 genes from the TGF-β signaling pathway, which has been shown as the most activated signaling pathway in the analysis of Li et al. (2017). Before reconstructing the gene regulatory network, we filter cell doublets and multiplets using an R package for single cell genomics, Seurat (Hao et al., 2021), and retain 472 cells, which contain ~ 40% zeros. To estimate the gene regulatory network, we use the HC algorithm for the nonlinear ZiG-DAG that assumes a zero-inflated hyper-Poisson distribution as the conditional distribution of each node. We initialize the algorithm with the DAG obtained by MRS, which shows promising performance in the experiments of Section 5.1.

**Figure 5:**
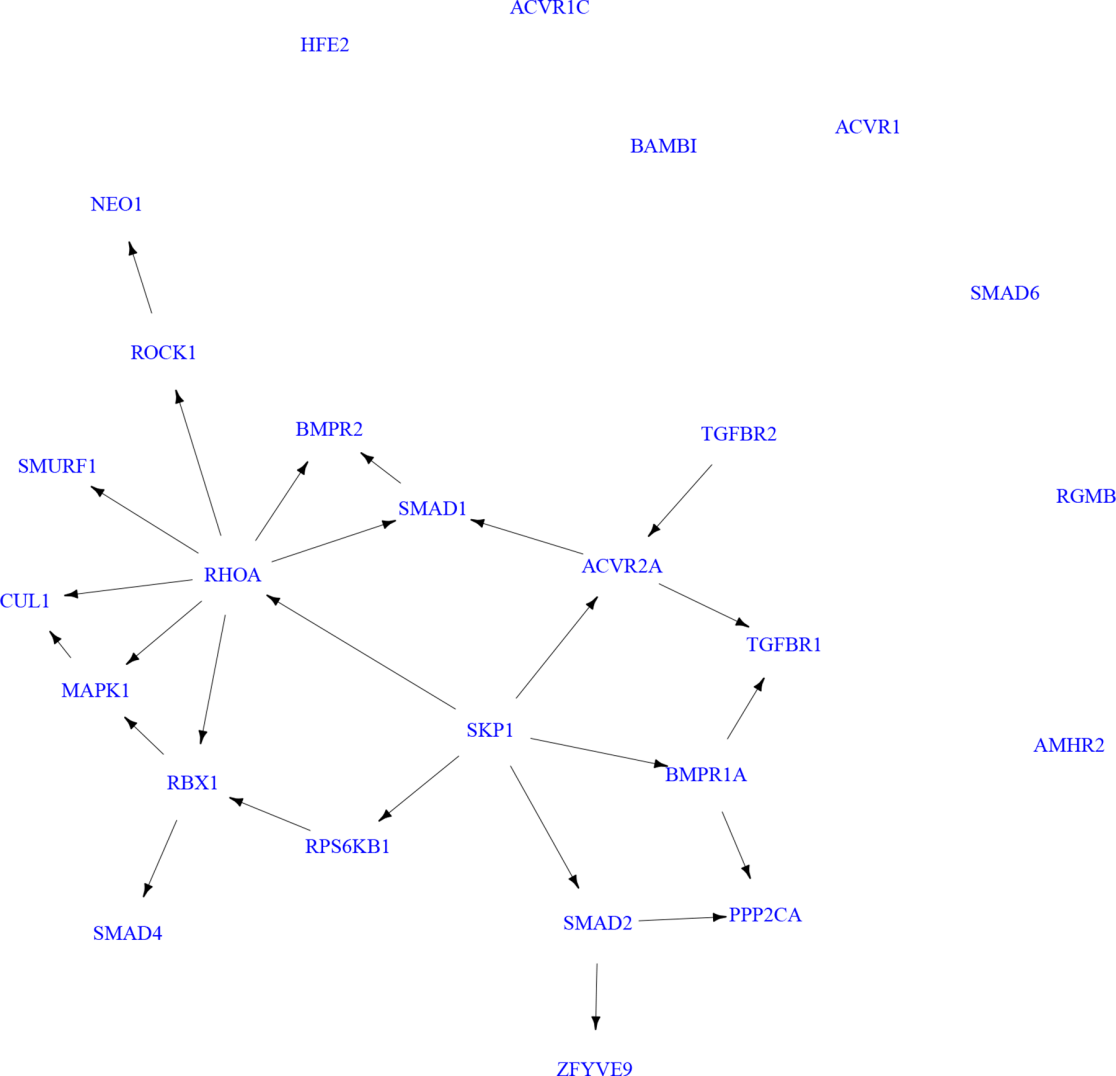
The estimated gene regulatory network for d=26 genes of the TGF-β signaling pathway using the nonlinear ZiG-DAGs.

Figure 5 displays the estimated gene regulatory network for d=26 genes of the TGF-β signaling pathway. In total, 26 directed edges are found by our nonlinear ZiG-DAG. Some of the estimated gene regulations are consistent with known regulatory relationships in the existing biological literature. For example, the proposed model finds gene regulations involving SMAD proteins, which are main signal transducers for receptors of the TGF-β superfamily. Specifically, SMAD2 affects mRNA profiles of ZFYVE9 (Runyan et al., 2009) and RBX1 regulates the SMAD4 protein stability (Inoue and Imamura, 2008). Moreover, the estimated network also confirms the fact that ROCK is a well-known downstream effector of RHOA.

Furthermore, we can find 2 hub genes in the estimated network: RHOA and SKP1 with out-degrees of 7 and 5. Hub genes are of particular importance because they are often involved in essential regulatory relationships. In fact, the importance of our hub genes in TGF-β signaling has been supported by the existing literature. RHOA is a small GTPase of the RHO family, whose inactivation plays key roles in colorectal cancer progression/metastasis by interacting with many members of TGF-β signaling pathway (Rodrigues et al., 2014; Dopeso et al., 2018). SKP1 belongs to the SCF complex, which is a RING-type E3 ubiquitin ligase that participates in the degradation of a wide variety of proteins that regulates TGF-β signaling (Inoue and Imamura, 2008).

## 7. Discussion

We have proposed a novel BN model, ZiG-DAG, to infer causal relationships in observational zero-inflated count data. ZiG-DAGs are built upon a fairly general class of count distributions, namely generalized hypergeometric probability distributions, and therefore can account for various types of zero-inflated count data including overdispersed or underdispersed zero-inflated count data. We have also considered not only linear causal relationships but also nonlinear relationships. The identifiability theory for the proposed ZiG-DAGs has been established using a general proof technique, which can potentially be used to show identifiability of other discrete BN models. The proposed ZiG-DAG models are paired with two structure learning procedures, exhaustive search and greedy search. Through extensive numerical experiments and real data analysis, we have empirically validated the identifiability theory for ZiG-DAGs and have shown its superior performance against state-of-the-art alternatives.

There are a few future research directions that can be taken. First, the proposed approach can be extended for modeling interventional zero-inflated count data. This may be done by modifying the likelihood according to the do-calculus framework of Pearl (2009). The second direction is to establish an identifiability theory in the presence of latent confounders. Although we have empirically shown in Section 5.4 that ZiG-DAG is relatively robust against confounding, we do not yet have theoretical support of it; the proofs of our identifiability theorems are not directly applicable as they rely on the factorization (1), which requires causal sufficiency. Third, the acyclicity of BNs may be restrictive in applications where the underlying systems have feedback loops, for example, genetic systems. We may relax this acyclicity restriction by using directed cyclic graphs.

## Figures and Tables

**Table 1:** Examples of GHPDs and their probability generating functions

Distributions	Probability generating function	Parameters
Binomial	F10(-n;;-ps/(1-p))F10(-n;;-p/(1-p))	0<p<1
Poisson	F00(;;θs)F00(;;θ)	θ>0
Hyper-Poisson	F11(1;ψ;θs)F11(1;ψ;θ)	ψ>0,θ>0
Geometric	F10(1;;qs)F10(1;;q)	0<q<1
Negative Binomial	F10(k;;qs)F10(k;;q)	k>0, 0<q<1
Hypergeometric	F21(-n,-Np;N-Np-n+1;s)F21(-n,-Np;N-Np-n+1;1)	n,N∈N,0<p<1
Beta-Negative Binomial	F21(k,ℓ;k+ℓ+m;s)F21(k,ℓ;k+ℓ+m;1)	k,ℓ,m≥0
Extended Generalized Waring	F21(k,ℓ;k+ℓ+m;θs)F21(k,ℓ;k+ℓ+m;θ)	k,ℓ>0,m∈R,0<θ<1

**Table 2:** Linear ZiG-DAG. Average operating characteristics over 50 simulations for different sample sizes n∈{250,500,1000,2000} with d=50. The standard error for each statistic is given within parentheses.

		Sample size, n			
Method	Measure	250	500	1000	2000
HC0	TPR	0.728 (0.009)	0.844 (0.009)	0.924 (0.008)	0.946 (0.007)
	FDR	0.387 (0.009)	0.226 (0.009)	0.125 (0.009)	0.083 (0.009)
	MCC	0.660 (0.008)	0.804 (0.009)	0.897 (0.009)	0.930 (0.008)
HC1	TPR	0.802 (0.009)	0.892 (0.007)	0.958 (0.005)	0.971 (0.004)
	FDR	0.354 (0.008)	0.203 (0.008)	0.101 (0.008)	0.074 (0.007)
	MCC	0.713 (0.008)	0.840 (0.007)	0.926 (0.007)	0.947 (0.005)
TS0	TPR	0.739 (0.009)	0.862 (0.009)	0.932 (0.007)	0.956 (0.006)
	FDR	0.391 (0.009)	0.222 (0.009)	0.121 (0.009)	0.074 (0.008)
	MCC	0.663 (0.009)	0.815 (0.008)	0.903 (0.008)	0.939 (0.007)
TS1	TPR	0.798 (0.009)	0.889 (0.008)	0.953 (0.006)	0.971 (0.004)
	FDR	0.369 (0.009)	0.214 (0.009)	0.110 (0.008)	0.073 (0.007)
	MCC	0.702 (0.009)	0.832 (0.008)	0.919 (0.007)	0.948 (0.005)
ODS	TPR	0.418 (0.006)	0.454 (0.004)	0.474 (0.005)	0.474 (0.003)
	FDR	0.710 (0.005)	0.726 (0.004)	0.753 (0.004)	0.776 (0.003)
	MCC	0.331 (0.005)	0.335 (0.004)	0.323 (0.004)	0.305 (0.003)
MRS	TPR	0.662 (0.006)	0.755 (0.004)	0.809 (0.004)	0.816 (0.003)
	FDR	0.467 (0.006)	0.454 (0.005)	0.464 (0.004)	0.505 (0.003)
	MCC	0.585 (0.006)	0.633 (0.004)	0.650 (0.004)	0.626 (0.003)
ZiDAG	TPR	0.619 (0.010)	0.710 (0.009)	0.756 (0.007)	0.778 (0.007)
	FDR	0.291 (0.010)	0.243 (0.009)	0.243 (0.008)	0.252 (0.008)
	MCC	0.656 (0.010)	0.727 (0.009)	0.751 (0.008)	0.758 (0.008)

**Table 3:** Linear ZiG-DAG. Average operating characteristics over 50 simulations for different numbers of nodes d∈{10,25,50,100} with n=1000. The standard error for each statistic is given within parentheses.

		Number of nodes, d			
Method	Measure	10	25	50	100
HC0	TPR	0.948 (0.012)	0.891 (0.013)	0.924 (0.008)	0.864 (0.007)
	FDR	0.067 (0.015)	0.166 (0.019)	0.125 (0.009)	0.255 (0.008)
	MCC	0.932 (0.015)	0.855 (0.017)	0.897 (0.009)	0.800 (0.007)
HC1	TPR	0.912 (0.014)	0.977 (0.005)	0.958 (0.005)	0.870 (0.006)
	FDR	0.141 (0.021)	0.060 (0.009)	0.101 (0.008)	0.269 (0.008)
	MCC	0.869 (0.020)	0.956 (0.007)	0.926 (0.007)	0.795 (0.007)
TS0	TPR	0.964 (0.010)	0.925 (0.011)	0.932 (0.007)	0.869 (0.006)
	FDR	0.051 (0.013)	0.130 (0.017)	0.121 (0.009)	0.254 (0.008)
	MCC	0.951 (0.013)	0.892 (0.015)	0.903 (0.008)	0.803 (0.007)
TS1	TPR	0.932 (0.014)	0.969 (0.005)	0.953 (0.006)	0.874 (0.007)
	FDR	0.103 (0.020)	0.081 (0.009)	0.110 (0.008)	0.267 (0.009)
	MCC	0.902 (0.019)	0.941 (0.007)	0.919 (0.007)	0.798 (0.008)
ODS	TPR	0.386 (0.010)	0.419 (0.008)	0.474 (0.005)	0.543 (0.004)
	FDR	0.677 (0.009)	0.775 (0.006)	0.753 (0.004)	0.761 (0.003)
	MCC	0.262 (0.010)	0.265 (0.007)	0.323 (0.004)	0.350 (0.003)
MRS	TPR	0.742 (0.010)	0.874 (0.009)	0.809 (0.004)	0.733 (0.004)
	FDR	0.331 (0.011)	0.423 (0.009)	0.464 (0.004)	0.623 (0.002)
	MCC	0.664 (0.010)	0.695 (0.009)	0.650 (0.004)	0.519 (0.003)
ZiDAG	TPR	0.640 (0.017)	0.700 (0.012)	0.756 (0.007)	0.794 (0.006)
	FDR	0.295 (0.016)	0.368 (0.016)	0.243 (0.008)	0.254 (0.007)
	MCC	0.632 (0.018)	0.649 (0.015)	0.751 (0.008)	0.768 (0.006)

**Table 4:** Nonlinear ZiG-DAG. Average operating characteristics over 50 simulations with n=500 and d=10. The standard error for each statistic is given within parentheses.

	Nonlinear ZiG-DAG	Linear ZiG-DAG	ODS	MRS	ZiDAG
TPR	0.622 (0.014)	0.662 (0.018)	0.614 (0.008)	0.588 (0.020)	0.568 (0.013)
FDR	0.179 (0.018)	0.377 (0.017)	0.417 (0.010)	0.405 (0.022)	0.249 (0.014)
MCC	0.684 (0.017)	0.596 (0.020)	0.546 (0.007)	0.540 (0.023)	0.616 (0.014)

**Table 5:** Non-zero-inflation. Average operating characteristics over 50 simulations for a negative binomial BN with n=500 and d=50. The standard error for each statistic is given within parentheses.

	ZiG-DAG-HP	ZiG-DAG-NB	ODS	MRS-HP	MRS-NB	ZiDAG
TPR	0.692 (0.009)	0.774 (0.007)	0.306 (0.004)	0.637 (0.008)	0.714 (0.008)	0.582 (0.008)
FDR	0.342 (0.010)	0.334 (0.008)	0.658 (0.005)	0.514 (0.009)	0.455 (0.009)	0.267 (0.010)
MCC	0.668 (0.009)	0.712 (0.007)	0.310 (0.004)	0.546 (0.008)	0.615 (0.008)	0.647 (0.009)

## Appendix A. Proof of Propostion 4

First, we state a lemma on the conditions under which conditional distributions of the same node are identical in two discrete BNs, which is needed for the proof of Proposition 4.

**Lemma 8** *Suppose that* ℬ=(𝒢,p) *and* ${ℬ}^{*}=\left({𝒢}^{*},p^{*}\right)$ *are any two discrete BNs, and* (1,2,…,d) *is the topological ordering of the DAG* 𝒢 *of* ℬ. *If for* $j^{\prime }\in V$, *we have* $pa\left(j^{\prime }\right)=pa^{*}\left(j^{\prime }\right),ch^{*}\left(j^{\prime }\right)\cap \text{nd}\left(j^{\prime }\right)=\emptyset$, *and*

(8)
$$\prod_{j=1}^{j^{\prime }}p\left(x_{j}|x_{pa(j)}\right)=\prod_{j=1}^{j^{\prime }}p^{*}\left(x_{j}|x_{pa^{*}(j)}\right),$$

*then the conditional distribution of node $j^{\prime }$ is the same in* ℬ *and* ${ℬ}^{*}$, *i.e.*, $p\left(x_{j^{\prime }}|x_{pa\left(j^{\prime }\right)}\right)=p^{*}\left(x_{j^{\prime }}|x_{pa^{*}\left(j^{\prime }\right)}\right)$.

**Proof** Since (1,…,d) is the topological ordering of 𝒢 and $ch^{*}\left(j^{\prime }\right)\cap \text{nd}\left(j^{\prime }\right)=\emptyset$, node $j^{\prime }$ cannot be a parent of nodes $\left\{1,2,\ldots ,j^{\prime }-1\right\}$ in both ℬ and ${ℬ}^{*}$, and hence in (8), $p\left(x_{j}|x_{pa(j)}\right)$ and $p^{*}\left(x_{j}|x_{pa^{*}(j)}\right)$ for $j=1,\ldots ,j^{\prime }-1$ are functions of all the variables $x_{1},\ldots ,x_{d}$ but $x_{j^{\prime }}$. The only terms in (8) that depends on $x_{j^{\prime }}$ are $p\left(x_{j^{\prime }}|x_{pa\left(j^{\prime }\right)}\right)$ and $p^{*}\left(x_{j^{\prime }}|x_{pa^{*}\left(j^{\prime }\right)}\right)$. Furthermore, both $p\left(x_{j^{\prime }}|x_{pa\left(j^{\prime }\right)}\right)$ and $p^{*}\left(x_{j^{\prime }}|x_{pa^{*}\left(j^{\prime }\right)}\right)$ are functions of $x_{j^{\prime }}$ and $x_{{pa}^{\cap }\left(j^{\prime }\right)}$, where we denote $pa^{\cap }(j)=pa\left(j^{\prime }\right)=pa^{*}\left(j^{\prime }\right)$. Let $x_{1},\ldots ,x_{j^{\prime }-1},x_{j^{\prime }+1},\ldots ,x_{d}$ be fixed. Then, (8) is simplified as

(9)
$$Cp\left(x_{j^{\prime }}|x_{pa\left(j^{\prime }\right)}\right)=C^{*}p^{*}\left(x_{j^{\prime }}|x_{pa^{*}\left(j^{\prime }\right)}\right)$$

where $C=\prod_{j=1}^{j^{\prime }-1}p\left(x_{j}|x_{pa(j)}\right)$ and $C^{*}=\prod_{j=1}^{j^{\prime }-1}p^{*}\left(x_{j}|x_{pa^{*}(j)}\right)$ are constants not depending on $x_{j^{\prime }}$. If we sum up (9) over all possible $x_{j^{\prime }}$, we have

$$C\sum_{x_{j^{\prime }}}p\left(x_{j^{\prime }}|x_{pa\left(j^{\prime }\right)}\right)=C^{*}\sum_{x_{j^{\prime }}}p^{*}\left(x_{j^{\prime }}|x_{pa^{*}\left(j^{\prime }\right)}\right).$$

Due to the fact that $\sum_{x_{j^{\prime }}}p\left(x_{j^{\prime }}|x_{pa\left(j^{\prime }\right)}\right)=1$ and $\sum_{x_{j^{\prime }}}p^{*}\left(x_{j^{\prime }}|x_{pa^{*}\left(j^{\prime }\right)}\right)=1$, we obtain $C=C^{*}$, and since $x_{1},\ldots ,x_{d}$ are arbitrary, it follows that $p\left(x_{j^{\prime }}|x_{pa\left(j^{\prime }\right)}\right)=p^{*}\left(x_{j^{\prime }}|x_{pa^{*}\left(j^{\prime }\right)}\right)$ for any possible $x_{j^{\prime }}$ and $x_{pa^{\cap }\left(j\right)}$.■

We now provide the proof of Proposition 4. We show that if the joint distributions p and $p^{*}$ are equivalent, then the identity of the causal structures 𝒢 and ${𝒢}^{*}$ automatically follows from the proposition assumption.

**Proof** We assume that the joint distributions of ℬ and ${ℬ}^{*}$ are the same, i.e.,

(10)
$$\prod_{j=1}^{d}p\left(x_{j}|x_{pa(j)}\right)=\prod_{j=1}^{d}p^{*}\left(x_{j}|x_{pa^{*}(j)}\right)$$

for all possible values of $x_{1},\ldots ,x_{d}$. Without loss of generality, assume that (1,…,d) is the topological ordering of the DAG 𝒢 of ℬ, i.e., the nodes are labeled such that there is no directed edge in 𝒢 from later nodes to earlier nodes. Such orderings must exist (although not necessarily unique) because of the acyclicity of DAGs. We then show by mathematical induction that $pa(j)=pa^{*}(j)$ for all j=1,2,…,d (hence $E=E^{*}$), which contradicts our assumption that $E\neq E^{*}$.

We begin with the last node d that has no child in the graph 𝒢. Taking the ratio of (10) at $x_{d}+1$ and $x_{d}$, we obtain

$$\frac{p\left(x_{d}+1|x_{pa(d)}\right)}{p\left(x_{d}|x_{pa(d)}\right)}=\frac{p^{*}\left(x_{d}+1|x_{pa^{*}(d)}\right)}{p^{*}\left(x_{d}|x_{pa^{*}(d)}\right)}\prod_{k\in ch^{*}(d)}\frac{p^{*}\left(x_{k}|x_{pa^{*}(k)\setminus \{d\}},x_{d}+1\right)}{p^{*}\left(x_{k}|x_{pa^{*}(k)\setminus \{d\}},x_{d}\right)}$$

for all $x_{1},\ldots ,x_{d}$. Note that this implicitly assumes that the conditional distributions $p\left(x_{j}|x_{pa(j)}\right)$ and $p^{*}\left(x_{j}|x_{pa^{*}(j)}\right),j=1,\ldots ,p$, are positive over their supports. The above ratio can be rewritten using probability generating functions as follows:

$$\frac{G_{d}^{\left(x_{d}+1\right)}\left(0;x_{pa(d)}\right)}{G_{d}^{\left(x_{d}\right)}\left(0;x_{pa(d)}\right)}=\frac{G_{d}^{*\left(x_{d}+1\right)}\left(0;x_{pa^{*}(d)}\right)}{G_{d}^{*\left(x_{d}\right)}\left(0;x_{pa^{*}(d)}\right)}\prod_{k\in ch^{*}(d)}\frac{G_{k}^{*\left(x_{k}\right)}\left(0;x_{pa^{*}(k)\setminus \{d\}},x_{d}+1\right)}{G_{k}^{*\left(x_{k}\right)}\left(0;x_{pa^{*}(k)\setminus \{d\}},x_{d}\right)}.$$

The proposition assumption then indicates $pa(d)=pa^{*}(d)$ and $ch^{*}(d)=ch^{*}(d)\cap \text{nd}(d)=\emptyset$. Moreover, Lemma 8 implies that $p\left(x_{d}|x_{pa(d)}\right)=p^{*}\left(x_{d}|x_{pa^{*}(d)}\right)$.

Now assume that for any $j=j^{\prime }+1,\ldots ,d$, it holds that $pa(j)=pa^{*}(j)$, $ch^{*}(j)\cap \text{nd}(j)=\emptyset$ and $p\left(x_{j}|x_{pa(j)}\right)=p^{*}\left(x_{j}|x_{pa^{*}(j)}\right)$. We then show that it also holds for $j=j^{\prime }$. First, observe that the ratio of (10) at $x_{j^{\prime }}+1$ and $x_{j^{\prime }}$ is given by

(11)
$$\frac{p\left(x_{j^{\prime }}+1|x_{pa\left(j^{\prime }\right)}\right)}{p\left(x_{j^{\prime }}|x_{pa\left(j^{\prime }\right)}\right)}\prod_{k\in ch\left(j^{\prime }\right)}\frac{p\left(x_{k}|x_{pa(k)\setminus \left\{j^{\prime }\right\}},x_{j^{\prime }}+1\right)}{p\left(x_{k}|x_{pa(k)\setminus \left\{j^{\prime }\right\}},x_{j^{\prime }}\right)}\;=\frac{p^{*}\left(x_{j^{\prime }}+1|x_{pa^{*}\left(j^{\prime }\right)}\right)}{p^{*}\left(x_{j^{\prime }}|x_{pa^{*}\left(j^{\prime }\right)}\right)}\prod_{k\in ch^{*}\left(j^{\prime }\right)}\frac{p^{*}\left(x_{k}|x_{pa^{*}(k)\setminus \left\{j^{\prime }\right\}},x_{j^{\prime }}+1\right)}{p^{*}\left(x_{k}|x_{pa^{*}(k)\setminus \left\{j^{\prime }\right\}},x_{j^{\prime }}\right)}.$$

Note that the induction assumption implies $ch\left(j^{\prime }\right)={ch}^{*}\left(j^{\prime }\right)\setminus \text{nd}\left(j^{\prime }\right)$ and

$$\frac{p\left(x_{k}|x_{pa(k)\setminus \left\{j^{\prime }\right\}},x_{j^{\prime }}+1\right)}{p\left(x_{k}|x_{pa(k)\setminus \left\{j^{\prime }\right\}},x_{j^{\prime }}\right)}=\frac{p^{*}\left(x_{k}|x_{pa^{*}(k)\setminus \left\{j^{\prime }\right\}},x_{j^{\prime }}+1\right)}{p^{*}\left(x_{k}|x_{pa^{*}(k)\setminus \left\{j^{\prime }\right\}},x_{j^{\prime }}\right)}$$

for $k\in ch\left(j^{\prime }\right)={ch}^{*}\left(j^{\prime }\right)\setminus \text{nd}\left(j^{\prime }\right)$. Therefore, we can simplify (11) into a similar form of the case j=d:

$$\frac{G_{j^{\prime }}^{\left(x_{j^{\prime }}+1\right)}\left(0;x_{pa\left(j^{\prime }\right)}\right)}{G_{j^{\prime }}^{\left(x_{j^{\prime }}\right)}\left(0;x_{pa\left(j^{\prime }\right)}\right)}=\frac{G_{j^{\prime }}^{*\left(x_{j^{\prime }}+1\right)}\left(0;x_{pa^{*}\left(j^{\prime }\right)}\right)}{G_{j^{\prime }}^{*\left(x_{j^{\prime }}\right)}\left(0;x_{pa^{*}\left(j^{\prime }\right)}\right)}\prod_{k\in ch^{*}\left(j^{\prime }\right)\cap \text{nd}\left(j^{\prime }\right)}\frac{G_{k}^{*\left(x_{k}\right)}\left(0;x_{pa^{*}(k)\setminus \left\{j^{\prime }\right\}},x_{j^{\prime }}+1\right)}{G_{k}^{*\left(x_{k}\right)}\left(0;x_{pa^{*}(k)\setminus \left\{j^{\prime }\right\}},x_{j^{\prime }}\right)}.$$

It again follows from the proposition assumption and Lemma 8 that $pa\left(j^{\prime }\right)=pa^{*}\left(j^{\prime }\right),ch^{*}\left(j^{\prime }\right)\cap \text{nd}\left(j^{\prime }\right)=\emptyset$, and $p\left(x_{j^{\prime }}|x_{pa\left(j^{\prime }\right)}\right)=p^{*}\left(x_{j^{\prime }}|x_{pa^{\prime }\left(j^{\prime }\right)}\right)$, which completes the proof.■

## Appendix B. Proof of Corollary 5

**Proof** Due to Proposition 4 and its assumption, the equivalence of the joint distributions of ℬ and ${ℬ}^{*}$ (i.e., $p=p^{*}$) implies $E=E^{*}$, which in turn implies $p\left(x_{j}|x_{pa(j)}\right)=p^{*}\left(x_{j}|x_{pa^{*}(j)}\right)$ for any j∈V. Then by the corollary assumption, $\Xi_{j}=\Xi_{j}^{*}$ for any j∈V. Hence, $\Xi =\Xi^{*}$.■

## Appendix C. Proof of Theorem 6

**Proof** We use Proposition 4 to prove that the DAG is identifiable for the linear ZiG-DAGs. Furthermore, we use Corollary 5 to show that the model parameters are also identifiable up to permutations of $a_{j}$ and $b_{j}$, given that ($p_{j},q_{j}$), which characterize the generalized hypergeometric function, and the link function $h_{j}$ are fixed for each j∈V. Let ℬ and ${ℬ}^{*}$ be two arbitrary linear ZiG-DAGs and we show that they satisfy the sufficient condition of Proposition 4. Let j∈V be a node such that the identity (6) holds. We show that $pa(j)=pa^{*}(j)$ and $ch^{*}(j)\cap \text{nd}(j)=\emptyset$. We use the superscript * to indicate parameters that define the linear ZiG-DAG ${ℬ}^{*}$.

First, we show $ch^{*}(j)\cap \text{nd}(j)=\emptyset$. Suppose by way of contradiction that $h^{*}(j)\cap \text{nd}(j)\neq \emptyset$, and let $k^{\prime }\in ch^{*}(j)\cap \text{nd}(j)$ such that $ch^{*}\left(k^{\prime }\right)\cap ch^{*}(j)\cap \text{nd}(j)=\emptyset$; such $k^{\prime }$ always exists due to the acyclicity of ${𝒢}^{*}$. For (6), let $x_{j}=0$ while fixing $x_{l}$ for $l\in V\setminus \left\{j,k^{\prime }\right\}$. Then, if $k^{\prime }\notin pa(j)$, it is simplified to

$$C_{1}=\left\{\begin{array}{cc} C_{2}\frac{1+\text{exp}{\left(\alpha_{k^{\prime }j}^{*}+{\tilde{\delta }}_{k^{\prime }}^{*}\right)}_{p_{k^{\prime }}^{*}}F_{p_{k^{\prime }}^{*}}\left(a_{k^{\prime }}^{*};b_{k^{\prime }}^{*};H_{k^{\prime }}^{*}\left(\beta_{k^{\prime }j}^{*}+{\tilde{\gamma }}_{k^{\prime }}^{*}\right)\right)}{1+\text{exp}{\left({\tilde{\delta }}_{k^{\prime }}^{*}\right)}_{p_{k^{\prime }}^{*}}F_{p_{k^{\prime }}^{*}}\left(a_{k^{\prime }}^{*};b_{k^{\prime }}^{*};H_{k^{\prime }}^{*}\left({\tilde{\gamma }}_{k^{\prime }}^{*}\right)\right)} & \text{for}\;x_{k^{\prime }}=0\\ C_{2}r^{x_{k^{\prime }}} & \text{for}\;x_{k^{\prime }}\neq 0 \end{array}\right.$$

and if $k^{\prime }\in pa(j)$, it takes the following form:

$$\frac{C_{3}H_{j}\left(\beta_{jk^{\prime }}x_{k^{\prime }}+{\tilde{\gamma }}_{j}\right)}{1+\text{exp}{\left(\alpha_{jk^{\prime }}x_{k^{\prime }}+{\tilde{\delta }}_{j}\right)}_{p_{j}}F_{q_{j}}\left(a_{j};b_{j};H_{j}\left(\beta_{jk^{\prime }}x_{k^{\prime }}+{\tilde{\gamma }}_{j}\right)\right)}\;=\left\{\begin{array}{ll} C_{2}\frac{\left.1+\text{exp}{\left(\alpha_{k^{\prime }j}^{\text{*}}+\delta_{k^{\prime }}^{\text{*}}\right)}_{p_{k^{\prime }}^{\text{*}}}F_{p_{k^{\prime }}^{\text{*}}}\left(a_{k^{\prime }}^{\text{*}};b_{k^{\prime }}^{\text{*}};H_{k^{\prime }}^{\text{*}}\left(\beta_{k^{\prime }j}^{\text{*}}+\gamma_{k^{\prime }}^{\text{*}}\right)\right)\right)}{\left.1+\text{exp}{\left(\delta_{k^{\prime }}^{\text{*}}\right)}_{p_{k^{\prime }}^{\text{*}}}F_{p_{k^{\prime }}^{\text{*}}}\left(a_{k^{\prime }}^{\text{*}};b_{k^{\prime }}^{\text{*}};H_{k^{\prime }}^{\text{*}}\left(\gamma_{k^{\prime }}^{\text{*}}\right)\right)\right)} & \text{for}\;x_{k^{\prime }}=0\\ C_{2}r^{x_{k^{\prime }}} & \text{for}\;x_{k^{\prime }}\neq 0, \end{array}\right.$$

where $H_{j}=h_{j}^{-1},H_{k^{\prime }}^{*}={\left(h_{k^{\prime }}^{*}\right)}^{-1},{\tilde{\delta }}_{j}=\sum_{l\in pa(j)\setminus \left\{k^{\prime }\right\}}\alpha_{jl}x_{l}+\delta_{j},{\tilde{\gamma }}_{j}=\sum_{l\in pa(j)\setminus \left\{k^{\prime }\right\}}\beta_{jl}x_{l}+\gamma_{j},{\tilde{\delta }}_{k^{\prime }}^{*}=\sum_{l\in pa^{*}\left(k^{\prime }\right)\setminus \{j\}}\alpha_{k^{\prime }l}^{*}x_{l}+\delta_{k^{\prime }}^{*},{\tilde{\gamma }}_{k^{\prime }}^{*}=\sum_{l\in pa^{*}\left(k^{\prime }\right)\setminus \{j\}}\beta_{k^{\prime }l}^{*}x_{l}+\gamma_{k^{\prime }}^{*}$, and

$$r=\frac{H_{k^{\prime }}^{*}\left(\beta_{k^{\prime }j}^{*}+{\tilde{\gamma }}_{k^{\prime }}^{*}\right)}{H_{k^{\prime }}^{*}\left({\tilde{\gamma }}_{k^{\prime }}^{*}\right)}.$$

Here, $C_{1},C_{2},C_{3}$ are some constants not depending on $x_{k^{\prime }}$. The above identities are well defined since each conditional distribution (or equivalently, the derivatives of the probability generating function) should be positive over the entire support in our definition of the linear ZiG-DAG. Since $x_{k^{\prime }}$ is not a binary variable (i.e., it takes integers beyond {0, 1}), taking the ratio of each of the above equations at $x_{k^{\prime }}+1$ and $x_{k^{\prime }}$, we observe that if $k^{\prime }\notin pa(j)$,

$$r=\frac{1+\text{exp}{\left(\alpha_{k^{\prime }j}^{*}+{\tilde{\delta }}_{k^{\prime }}^{*}\right)}_{p_{k^{\prime }}^{*}}F_{p_{k^{\prime }}^{*}}\left(a_{k^{\prime }}^{*};b_{k^{\prime }}^{*};H_{k^{\prime }}^{*}\left(\beta_{k^{\prime }j}^{*}+{\tilde{\gamma }}_{k^{\prime }}^{*}\right)\right)}{1+\text{exp}{\left({\tilde{\delta }}_{k^{\prime }}^{*}\right)}_{p_{k^{\prime }}^{*}}F_{p_{k^{\prime }}^{*}}\left(a_{k^{\prime }}^{*};b_{k^{\prime }}^{*};H_{k^{\prime }}^{*}\left({\tilde{\gamma }}_{k^{\prime }}^{*}\right)\right)}=1;$$

and if $k^{\prime }\in pa(j)$,

$$r=\frac{1+\text{exp}{\left(\alpha_{k^{\prime }j}^{*}+{\tilde{\delta }}_{k^{\prime }}^{*}\right)}_{p_{k^{\prime }}^{*}}F_{p_{k^{\prime }}^{*}}\left(a_{k^{\prime }}^{*};b_{k^{\prime }}^{*};H_{k^{\prime }}^{*}\left(\beta_{k^{\prime }j}^{*}+{\tilde{\gamma }}_{k^{\prime }}^{*}\right)\right)}{1+\text{exp}{\left({\tilde{\delta }}_{k^{\prime }}^{*}\right)}_{p_{k^{\prime }}^{*}}^{*}F_{p_{k^{\prime }}^{*}}^{*}\left(a_{k^{\prime }}^{*};b_{k^{\prime }}^{*};H_{k^{\prime }}^{*}\left({\tilde{\gamma }}_{k^{\prime }}^{*}\right)\right)}\;\times \frac{H_{j}\left(\beta_{jk^{\prime }}+{\tilde{\gamma }}_{j}\right)\left\{1+\text{exp}{\left({\tilde{\delta }}_{j}\right)}_{p_{j}}F_{q_{j}}\left(a_{j};b_{j};H_{j}\left({\tilde{\gamma }}_{j}\right)\right)\right\}}{H_{j}\left({\tilde{\gamma }}_{j}\right)\left\{1+\text{exp}{\left(\alpha_{jk^{\prime }}+{\tilde{\delta }}_{j}\right)}_{p_{j}}F_{q_{j}}\left(a_{j};b_{j};H_{j}\left(\beta_{jk^{\prime }}+{\tilde{\gamma }}_{j}\right)\right)\right\}}$$

and

$$r=\frac{H_{j}\left(\beta_{jk^{\prime }}\left(x_{k^{\prime }}+1\right)+{\tilde{\gamma }}_{j}\right)\left\{1+\text{exp}{\left(\alpha_{jk^{\prime }}x_{k^{\prime }}+{\tilde{\delta }}_{j}\right)}_{p_{j}}F_{q_{j}}\left(a_{j};b_{j};H_{j}\left(\beta_{jk^{\prime }}x_{k^{\prime }}+{\tilde{\gamma }}_{j}\right)\right)\right\}}{H_{j}\left(\beta_{jk^{\prime }}x_{k^{\prime }}+{\tilde{\gamma }}_{j}\right)\left\{1+\text{exp}{\left(\alpha_{jk^{\prime }}\left(x_{k^{\prime }}+1\right)+{\tilde{\delta }}_{j}\right)}_{p_{j}}F_{q_{j}}\left(a_{j};b_{j};H_{j}\left(\beta_{jk^{\prime }}\left(x_{k^{\prime }}+1\right)+{\tilde{\gamma }}_{j}\right)\right)\right\}}$$

for any possible positive value of $x_{k^{\prime }}$ in the support of $X_{k^{\prime }}$. Since these equations hold for all possible values of $x_{l},l\in V\setminus \left\{j,k^{\prime }\right\}$, in both cases, we have that r=1 and $\alpha_{k^{\prime }j}^{*}=\beta_{k^{\prime }j}^{*}=0$. This indicates $k^{\prime }\notin ch^{*}(j)$, which contradicts the assumption that $k^{\prime }\in ch^{*}(j)\cap \text{nd}(j)(\neq \emptyset )$.

We now show $pa(j)=pa^{*}(j)$. Given the above result, $ch^{*}(j)\cap \text{nd}(j)=\emptyset$, we can simplify (6) as

(12)
$$\frac{\left(a_{j1}+x_{j}\right)\cdots \left(a_{jp_{j}}+x_{j}\right)H_{j}\left(\sum_{k\in pa(j)}\beta_{\text{jk}}x_{k}+\gamma_{j}\right)}{\left(b_{j1}+x_{j}\right)\cdots \left(b_{jq_{j}}+x_{j}\right)\left(x_{j}+1\right)}\;=\frac{\left(a_{j1}^{*}+x_{j}\right)\cdots \left(a_{jp_{j}^{*}}^{*}+x_{j}\right)H_{j}^{*}\left(\sum_{k\in pa^{*}(j)}\beta_{\text{jk}}^{*}x_{k}+\gamma_{j}^{*}\right)}{\left(b_{j1}^{*}+x_{j}\right)\cdots \left(b_{jq_{j}^{*}}^{*}+x_{j}\right)\left(x_{j}+1\right)}.$$

for a positive integer $x_{j}$. For $l\in pa(j)\setminus pa^{*}(j)$, taking the ratio of (12) at $x_{l}=1$ and $x_{l}=0$, we obtain

$$\frac{H_{j}\left(\sum_{k\in pa(j)\setminus \{l\}}\beta_{\text{jk}}x_{k}+\beta_{jl}+\gamma_{j}\right)}{H_{j}\left(\sum_{k\in pa(j)\setminus \{l\}}\beta_{\text{jk}}x_{k}+\gamma_{j}\right)}=1,$$

which leads to $\beta_{jl}=0$. Next, if $x_{j}=0$, (6) is simplified as

(13)
$$\frac{a_{j1}\cdots a_{jp_{j}}H_{j}\left(\sum_{k\in pa(j)}\beta_{\text{jk}}x_{k}+\gamma_{j}\right)}{b_{j1}\cdots b_{jq_{j}}\left\{1+\text{exp}{\left(\sum_{k\in pa(j)}\alpha_{\text{jk}}x_{k}+\delta_{j}\right)}_{p_{j}}F_{q_{j}}\left(a_{j};b_{j};H_{j}\left(\sum_{k\in pa(j)\cap pa^{*}(j)}\beta_{\text{jk}}x_{k}+\gamma_{j}\right)\right)\right\}}\;=\frac{a_{j1}^{*}\cdots a_{jp_{j}^{*}}^{*}H_{j}^{*}\left(\sum_{k\in pa^{*}(j)}\beta_{\text{jk}}^{*}x_{k}+\gamma_{j}^{*}\right)}{b_{j1}^{*}\cdots b_{jq_{j}^{*}}^{*}\left\{1+\text{exp}{\left(\sum_{k\in pa^{*}(j)}\alpha_{\text{jk}}^{*}x_{k}+\delta_{j}^{*}\right)}_{p_{j}^{*}}F_{q_{j}^{*}}\left(a_{j}^{*};b_{j}^{*};H_{j}^{*}\left(\sum_{k\in pa(j)\cap pa^{*}(j)}\beta_{\text{jk}}^{*}x_{k}+\gamma_{j}^{*}\right)\right)\right\}}.$$

Again, take the ratio of (13) at $x_{l}=1$ and $x_{l}=0$ for $l\in pa(j)\setminus pa^{*}(j)$. We have

$$\frac{1+\text{exp}{\left(\sum_{k\in pa(j)\setminus \{l\}}\alpha_{\text{jk}}x_{k}+\alpha_{jl}+\delta_{j}\right)}_{p_{j}}F_{q_{j}}\left(a_{j};b_{j};H_{j}\left(\sum_{k\in pa(j)\cap pa^{*}(j)}\beta_{\text{jk}}x_{k}+\gamma_{j}\right)\right)}{1+\text{exp}{\left(\sum_{k\in pa(j)\setminus \{l\}}\alpha_{\text{jk}}x_{k}+\delta_{j}\right)}_{p_{j}}F_{q_{j}}\left(a_{j};b_{j};H_{j}\left(\sum_{k\in pa(j)\cap pa^{*}(j)}\beta_{\text{jk}}x_{k}+\gamma_{j}\right)\right)}=1,$$

and therefore $\alpha_{jl}=0$. The finding that $\alpha_{jl}=\beta_{jl}=0$ for $l\in pa(j)\setminus pa^{*}(j)$ implies that $pa(j)\setminus pa^{*}(j)=\emptyset$. Similarly, we get $pa^{*}(j)\setminus pa(j)=\emptyset$, and hence $pa(j)=pa^{*}(j)$.

Next, we show that the model parameters of the linear ZiG-DAGs are also identifiable (up to permutations of $a_{j}$ and $b_{j}$), under the assumption that $p_{j},q_{j}$, and $h_{j}$ are fixed (i.e., $p_{j}=p_{j}^{*},q_{j}=q_{j}^{*}$, and $h_{j}=h_{j}^{*}$). We already know $pa(j)=pa^{*}(j)$ and thus we denote $pa^{\cap }(j)=pa(j)=pa^{*}(j)$. According to Corollary 5, it suffices to show that $p\left(x_{j}|x_{pa(j)}\right)=p^{*}\left(x_{j}|x_{pa^{*}(j)}\right)$ implies that $\alpha_{\text{jk}}=\alpha_{\text{jk}}^{*},\beta_{\text{jk}}=\beta_{\text{jk}}^{*}$ for $k\in pa^{n}(j)$, $\delta_{j}=\delta_{j}^{*},\gamma_{j}=\gamma_{j}^{*}$, and $a_{j}$ and $a_{j}^{*}$, as well as $b_{j}$ and $b_{j}^{*}$, are equivalent up to a permutation. If we take the ratio of $p\left(x_{j}|x_{pa(j)}\right)=p^{*}\left(x_{j}|x_{pa^{*}(j)}\right)$ at $x_{j}+1$ and $x_{j}$, we observe that

(14)
$$\frac{\left(a_{j1}+x_{j}\right)\cdots \left(a_{jp_{j}}+x_{j}\right)H_{j}\left(\sum_{k\in pa^{\cap }(j)}\beta_{\text{jk}}x_{k}+\gamma_{j}\right)}{\left(b_{j1}+x_{j}\right)\cdots \left(b_{jq_{j}}+x_{j}\right)\left(x_{j}+1\right)}\;=\frac{\left(a_{j1}^{*}+x_{j}\right)\cdots \left(a_{jp_{j}}^{*}+x_{j}\right)H_{j}\left(\sum_{k\in pa^{\cap }(j)}\beta_{\text{jk}}^{*}x_{k}+\gamma_{j}^{*}\right)}{\left(b_{j1}^{*}+x_{j}\right)\cdots \left(b_{jq_{j}}^{*}+x_{j}\right)\left(x_{j}+1\right)}$$

for $x_{j}\neq 0$, and

(15)
$$\frac{a_{j1}\cdots a_{jp_{j}}H_{j}\left(\sum_{k\in pa^{\cap }\left(j\right)}\beta_{jk}x_{k}+\gamma_{j}\right)}{b_{j1}\cdots b_{jq_{j}}\left\{1+\text{exp}{\left(\sum_{k\in pa^{\cap }\left(j\right)}\alpha_{jk}x_{k}+\delta_{j}\right)}_{p_{j}}F_{q_{j}}\left(a_{j};b_{j};H_{j}\left(\sum_{k\in pa^{\cap }\left(j\right)}\beta_{jk}x_{k}+\gamma_{j}\right)\right)\right\}}\;=\frac{a_{j1}^{*}\cdots a_{jp_{j}}^{*}H_{j}\left(\sum_{k\in pa^{\cap }\left(j\right)}\beta_{jk}^{*}x_{k}+\gamma_{j}^{*}\right)}{b_{j1}^{*}\cdots b_{jq_{j}}^{*}\left\{1+\text{exp}{\left(\sum_{k\in pa^{\cap }\left(j\right)}\alpha_{jk}^{*}x_{k}+\delta_{j}^{*}\right)}_{p_{j}}F_{q_{j}}\left(a_{j}^{*};b_{j}^{*};H_{j}\left(\sum_{k\in pa^{\cap }\left(j\right)}\beta_{jk}^{*}x_{k}+\gamma_{j}^{*}\right)\right)\right\}}$$

for $x_{j}=0$. Since (14) and (15) hold for any possible value of $x_{k}$ for $k\in pa^{\cap }(j)$, we must have $\alpha_{\text{jk}}=\alpha_{\text{jk}}^{*},\beta_{\text{jk}}=\beta_{\text{jk}}^{*}$ for $k\in pa^{\cap }(j)$, $\delta_{j}=\delta_{j}^{*}$, and $\gamma_{j}=\gamma_{j}^{*}$. It is also easy to see that $a_{j}$ and $a_{j}^{*}$, as well as $b_{j}$ and $b_{j}^{*}$, are equivalent up to a permutation.■

## Appendix D. Proof of Theorem 7

**Proof** To show that the graph structure of the nonlinear ZiG-DAGs is identifiable, we show $pa(j)=pa^{*}(j)$ and $ch^{*}(j)\cap \text{nd}(j)=\emptyset$, assuming the identity (6) holds for node j∈V for two arbitrary nonlinear ZiG-DAGs ℬ and ${ℬ}^{*}$. Additionally, in order to establish the parameter identifiability of the nonlinear ZiG-DAGs, we show that equivalence of the parameters follows $p\left(x_{j}|x_{pa(j)}\right)=p^{*}\left(x_{j}|x_{pa^{*}(j)}\right)$, under the assumption that for each $j\in V,p_{j},q_{j}$, and $h_{j}$ are fixed. We use the superscript * to indicate parameters that define the nonlinear ZiG-DAG ${ℬ}^{*}$.

We first show $ch^{*}(j)\cap \text{nd}(j)=\emptyset$. Suppose on the contrary that $ch^{*}(j)\cap \text{nd}(j)\neq \emptyset$. Consider $k^{\prime }\in ch^{*}(j)\cap \text{nd}(j)$ such that $ch^{*}\left(k^{\prime }\right)\cap ch^{*}(j)\cap \text{nd}(j)=\emptyset$, as in the proof of Theorem 6. Under the nonlinear ZiG-DAGs, letting $x_{j}=0$ while fixing $x_{l}$ for all $l\in V\setminus \left\{j,k^{\prime }\right\}$, we can simplify (6) as

$$C_{1}=\left\{\begin{array}{ll} C_{2}\frac{1+\text{exp}{\left(f_{k^{\prime }j}^{*}\left(1\right)+{\tilde{\mu }}_{k^{\prime }}^{*}\right)}_{p_{k^{\prime }}^{*}}F_{p_{k^{\prime }}^{*}}\left(a_{k^{\prime }}^{*};b_{k^{\prime }}^{*};H_{k^{\prime }}^{*}\left(g_{k^{\prime }j}^{*}\left(1\right)+{\tilde{\nu }}_{k^{\prime }}^{*}\right)\right)}{1+\text{exp}{\left(f_{k^{\prime }j}^{*}\left(0\right)+{\tilde{\mu }}_{k^{\prime }}^{*}\right)}_{p_{k^{\prime }}^{*}}F_{p_{k^{\prime }}^{*}}\left(a_{k^{\prime }}^{*};b_{k^{\prime }}^{*};H_{k^{\prime }}^{*}\left(g_{k^{\prime }j}^{*}\left(0\right)+{\tilde{\nu }}_{k^{\prime }}^{*}\right)\right)} & \text{for}\;x_{k^{\prime }}=0\\ C_{2}r^{x_{k^{\prime }}} & \text{for}\;x_{k^{\prime }}\neq 0, \end{array}\right.$$

if $k^{\prime }\notin pa(j)$, and

$$\frac{C_{3}H_{j}\left(g_{jk^{\prime }}\left(x_{k^{\prime }}\right)+{\tilde{\nu }}_{j}\right)}{1+\text{exp}{\left(f_{jk^{\prime }}\left(x_{k^{\prime }}\right)+{\tilde{\mu }}_{j}\right)}_{p_{j}}F_{q_{j}}\left(a_{j};b_{j};H_{j}\left(g_{jk^{\prime }}\left(x_{k^{\prime }}\right)+{\tilde{\nu }}_{j}\right)\right)}\;=\left\{\begin{array}{ll} C_{2}\frac{1+\text{exp}{\left(f_{k^{\prime }j}^{*}\left(1\right)+{\tilde{\mu }}_{k^{\prime }}^{*}\right)}_{p_{k^{\prime }}^{*}}F_{p_{k^{\prime }}^{*}}\left(a_{k^{\prime }}^{*};b_{k^{\prime }}^{*};H_{k^{\prime }}^{*}\left(g_{k^{\prime }j}^{*}\left(1\right)+{\tilde{\nu }}_{k^{\prime }}^{*}\right)\right)}{1+\text{exp}{\left(f_{k^{\prime }j}^{*}\left(0\right)+{\tilde{\mu }}_{k^{\prime }}^{*}\right)}_{p_{k^{\prime }}^{*}}F_{p_{k^{\prime }}^{*}}\left(a_{k^{\prime }}^{*};b_{k^{\prime }}^{*};H_{k^{\prime }}^{*}\left(g_{k^{\prime }j}^{*}\left(0\right)+{\tilde{\nu }}_{k^{\prime }}^{*}\right)\right)} & \text{for}\;x_{k^{\prime }}=0\\ C_{2}r^{x_{k^{\prime }}} & \text{for}\;x_{k^{\prime }}\neq 0, \end{array}\right.$$

if $k^{\prime }\in pa(j)$, where $C_{1},C_{2},C_{3}$ are some constants, $H_{j}=h_{j}^{-1},H_{k^{\prime }}^{*}={\left(h_{k^{\prime }}^{*}\right)}^{-1},{\tilde{\mu }}_{j}=\sum_{l\in pa(j)\setminus \left\{k^{\prime }\right\}}f_{jl}\left(x_{l}\right)+\mu_{j},{\tilde{\nu }}_{j}=\sum_{l\in pa(j)\setminus \left\{k^{\prime }\right\}}g_{jl}\left(x_{l}\right)+\nu_{j},{\tilde{\mu }}_{k^{\prime }}^{*}=\sum_{l\in pa^{*}\left(k^{\prime }\right)\setminus \{j\}}f_{k^{\prime }l}^{*}\left(x_{l}\right)+\mu_{k^{\prime }}^{*},{\tilde{\nu }}_{k^{\prime }}^{*}=\sum_{l\in pa^{*}\left(k^{\prime }\right)\setminus \{j\}}g_{k^{\prime }l}^{*}\left(x_{l}\right)+\nu_{k^{\prime }}^{*}$, and $r=\frac{H_{k^{\prime }}^{*}\left(g_{k^{\prime }j}^{*}(1)+{\tilde{\nu }}_{k^{\prime }}^{*}\right)}{H_{k^{\prime }}^{*}\left(g_{k^{\prime }j}^{*}(0)+{\tilde{\nu }}_{k^{\prime }}^{*}\right)}$. The above identities are well defined, because in our definition of the nonlinear ZiG-DAG, each conditional distribution (or equivalently, the derivatives of the probability generating function) should be positive over the entire support.

Because $x_{k^{\prime }}$ is not binary (i.e., it takes integers beyond {0, 1}), if we take the ratio of the first equation above at $x_{k^{\prime }}+1$ and $x_{k^{\prime }}$, we obtain that if $k^{\prime }\notin pa(j)$,

$$r=\frac{\left.\left.1+\text{exp}{\left(f_{k^{\prime }j}^{*}\left(1\right)+{\tilde{\mu }}_{k^{\prime }}^{*}\right)}_{p_{k^{\prime }}^{*}}F_{p_{k^{\prime }}^{*}}\left(a_{k^{\prime }}^{*};b_{k^{\prime }}^{*};H_{k^{\prime }}^{*}\left(g_{k^{\prime }j}^{*}\left(1\right)+{\tilde{\nu }}_{k^{\prime }}^{*}\right)\right)\right)\right)}{\left.\left.1+\text{exp}{\left(f_{k^{\prime }j}^{*}\left(0\right)+{\tilde{\mu }}_{k^{\prime }}^{*}\right)}_{p_{k^{\prime }}^{*}}F_{p_{k^{\prime }}^{*}}\left(a_{k^{\prime }}^{*};b_{k^{\prime }}^{*};H_{k^{\prime }}^{*}\left(g_{k^{\prime }j}^{*}\left(0\right)+{\tilde{\nu }}_{k^{\prime }}^{*}\right)\right)\right)\right)}=1.$$

If $k^{\prime }\in pa(j)$, we take the ratio of the second equation and obtain

$$r=\frac{\left.\left.1+\text{exp}{\left(f_{k^{\prime }j}^{*}\left(1\right)+{\tilde{\mu }}_{k^{\prime }}^{*}\right)}_{p_{k^{\prime }}^{*}}F_{p_{k^{\prime }}^{*}}\left(a_{k^{\prime }}^{*};b_{k^{\prime }}^{*};H_{k^{\prime }}^{*}\left(g_{k^{\prime }j}^{*}\left(1\right)+{\tilde{\nu }}_{k^{\prime }}^{*}\right)\right)\right)\right)}{\left.\left.1+\text{exp}{\left(f_{k^{\prime }j}^{*}\left(0\right)+{\tilde{\mu }}_{k^{\prime }}^{*}\right)}_{p_{k^{\prime }}^{*}}F_{p_{k^{\prime }}^{*}}\left(a_{k^{\prime }}^{*};b_{k^{\prime }}^{*};H_{k^{\prime }}^{*}\left(g_{k^{\prime }j}^{*}\left(0\right)+{\tilde{\nu }}_{k^{\prime }}^{*}\right)\right)\right)\right)}\;\times \frac{H_{j}\left(g_{jk^{\prime }}\left(1\right)+{\tilde{\nu }}_{j}\right)\left\{1+\text{exp}{\left(f_{jk^{\prime }}\left(0\right)+{\tilde{\mu }}_{j}\right)}_{p_{j}}F_{q_{j}}\left(a_{j};b_{j};H_{j}\left(g_{jk^{\prime }}\left(0\right)+{\tilde{\nu }}_{j}\right)\right)\right\}}{H_{j}\left(g_{jk^{\prime }}\left(0\right)+{\tilde{\nu }}_{j}\right)\left\{1+\text{exp}{\left(f_{jk^{\prime }}\left(1\right)+{\tilde{\mu }}_{j}\right)}_{p_{j}}F_{q_{j}}\left(a_{j};b_{j};H_{j}\left(g_{jk^{\prime }}\left(1\right)+{\tilde{\nu }}_{j}\right)\right)\right\}}$$

and

$$r=\frac{H_{j}\left(g_{jk^{\prime }}\left(x_{k^{\prime }}+1\right)+{\tilde{\nu }}_{j}\right)\left\{1+\text{exp}{\left(f_{jk^{\prime }}\left(x_{k^{\prime }}\right)+{\tilde{\mu }}_{j}\right)}_{p_{j}}F_{q_{j}}\left(a_{j};b_{j};H_{j}\left(g_{jk^{\prime }}\left(x_{k^{\prime }}\right)+{\tilde{\nu }}_{j}\right)\right)\right\}}{H_{j}\left(g_{jk^{\prime }}\left(x_{k^{\prime }}\right)+{\tilde{\nu }}_{j}\right)\left\{1+\text{exp}{\left(f_{jk^{\prime }}\left(x_{k^{\prime }}+1\right)+{\tilde{\mu }}_{j}\right)}_{p_{j}}F_{q_{j}}\left(a_{j};b_{j};H_{j}\left(g_{jk^{\prime }}\left(x_{k^{\prime }}+1\right)+{\tilde{\nu }}_{j}\right)\right)\right\}}$$

for any possible positive value of $x_{k^{\prime }}$ in the support of $X_{k^{\prime }}$. Similar to the proof of Theorem 6, we necessarily have in both cases that r=1 as well as $f_{k^{\prime }j}^{*}(0)=f_{k^{\prime }j}^{*}(1)$, $g_{k^{\prime }j}^{*}(0)=g_{k^{\prime }j}^{*}(1)$. Now, we consider the ratio of (6) at $x_{k^{\prime }}=1$ and $x_{k^{\prime }}=0$ with arbitrary $x_{j}\neq 0$. Observe that

$$\frac{1+\text{exp}{\left(f_{k^{\prime }j}^{*}\left(x_{j}\right)+{\tilde{\mu }}_{k^{\prime }}^{*}\right)}_{p_{k^{\prime }}^{*}}F_{q_{k^{\prime }}^{*}}\left(a_{k^{\prime }}^{*};b_{k^{\prime }}^{*};H_{k^{\prime }}\left(g_{k^{\prime }j}^{*}\left(x_{j}\right)+{\tilde{\nu }}_{k^{\prime }}^{*}\right)\right.}{1+\text{exp}{\left(f_{k^{\prime }j}^{*}\left(x_{j}+1\right)+{\tilde{\mu }}_{k^{\prime }}^{*}\right)}_{p_{k^{\prime }}^{*}}F_{q_{k^{\prime }}^{*}}\left(a_{k^{\prime }}^{*};b_{k^{\prime }}^{*};H_{k^{\prime }}\left(g_{k^{\prime }j}^{*}\left(x_{j}+1\right)+{\tilde{\nu }}_{k^{\prime }}^{*}\right)\right.}=1$$

holds for any $x_{j}\neq 0$, indicating $f_{k^{\prime }j}^{*}\left(x_{j}\right)=f_{k^{\prime }j}^{*}\left(x_{j}+1\right)$ and $g_{k^{\prime }j}^{*}\left(x_{j}\right)=g_{k^{\prime }j}^{*}\left(x_{j}+1\right)$ for all possible $x_{j}\neq 0$. All together, we have $f_{k^{\prime }j}^{*}\left(x_{j}\right)=f_{k^{\prime }j}^{*}\left(x_{j}+1\right)$ and $g_{k^{\prime }j}^{*}\left(x_{j}\right)=g_{k^{\prime }j}^{*}\left(x_{j}+1\right)$ for any possible value of $x_{j}$. Because we have assumed $E\left[f_{\text{jk}}^{*}\left(X_{k}\right)\right]=E\left[g_{\text{jk}}^{*}\left(X_{k}\right)\right]=0$ for all j,k, where $X_{k}$ are count random variables, it implies that $f_{k^{\prime }j}^{*}\left(x_{j}\right)=g_{k^{\prime }j}^{*}\left(x_{j}\right)=0$ for any value of $x_{j}$ in its support, and hence $k^{\prime }\notin ch^{*}(j)$. This contradicts the assumption that $k^{\prime }\in ch^{*}(j)\cap \text{nd}(j)(\neq \emptyset )$.

Next, we show $pa(j)=pa^{*}(j)$. Let $l\in pa(j)\setminus pa^{*}(j)$. Since $ch^{*}(j)\cap \text{nd}(j)=\emptyset$, by taking the ratio of (6) at $x_{l}+1$ and $x_{l}$, we have

$$\frac{r_{j}\left(x_{j};x_{l}+1,x_{pa(j)\setminus \{l\}}\right)}{r_{j}\left(x_{j};x_{l},x_{pa(j)\setminus \{l\}}\right)}=1$$

for all $x_{j}$ and $x_{pa(j)}$, where

$$r_{j}\left(x_{j};x_{pa(j)}\right)=\left\{\begin{array}{ll} \frac{a_{j1}\cdots a_{jp_{j}}H_{j}\left(\sum_{k\in pa(j)}g_{\text{jk}}\left(x_{k}\right)+\nu_{j}\right)}{b_{j1}\cdots b_{jq_{j}}\left\{1+\text{exp}{\left(\sum_{k\in pa(j)}f_{\text{jk}}\left(x_{k}\right)+\mu_{j}\right)}_{p_{j}}F_{q_{j}}\left(a_{j};b_{j};H_{j}\left(\sum_{k\in pa(j)}g_{\text{jk}}\left(x_{k}\right)+\nu_{j}\right)\right)\right\}} & \text{if}\;x_{j}=0\\ \frac{\left(a_{j1}+x_{j}\right)\cdots \left(a_{jp_{j}}+x_{j}\right)H_{j}\left(\sum_{k\in pa(j)}g_{\text{jk}}\left(x_{k}\right)+\nu_{j}\right)}{\left(b_{j1}+x_{j}\right)\cdots \left(b_{jq_{j}}+x_{j}\right)\left(x_{j}+1\right)} & \text{if}\;x_{j}\neq 0. \end{array}\right.$$

It easily follows that $f_{jl}\left(x_{l}+1\right)=f_{jl}\left(x_{l}\right)$ and $g_{jl}\left(x_{l}+1\right)=g_{jl}\left(x_{l}\right)$ for all possible values of $x_{l}$, and combining this again with the assumption that $E\left[f_{\text{jk}}^{*}\left(X_{k}\right)\right]=E\left[g_{\text{jk}}^{*}\left(X_{k}\right)\right]=0$, we have $pa^{*}(j)\setminus pa(j)=\emptyset$. Similarly, we obtain $pa^{*}(j)\setminus pa(j)=\emptyset$, which implies $pa(j)=pa^{*}(j)$.

Lastly, we show that if $p_{j},q_{j}$, and $h_{j}$ are fixed (i.e., $p_{j}=p_{j}^{*},q_{j}=q_{j}^{*}$, and $h_{j}=h_{j}^{*}$), we can deduce from $p\left(x_{j}|x_{pa(j)}\right)=p^{*}\left(x_{j}|x_{pa^{*}(j)}\right)$ that $\left(f_{\text{jk}},g_{\text{jk}}\right)=\left(f_{\text{jk}}^{*},g_{\text{jk}}^{*}\right)$ for $k\in pa^{\cap }(j)$, $\mu_{j}=\mu_{j}^{*},\nu_{j}=\nu_{j}^{*}$, and $a_{j}$ and $b_{j}$ are equivalent to $a_{j}^{*}$ and $b_{j}^{*}$ up to permutations. Here, we denote $pa^{\cap }(j)=pa(j)=pa^{*}(j)$ as in the proof of Theorem 6. Consider the ratio of $p\left(x_{j}|x_{pa(j)}\right)=p^{*}\left(x_{j}|x_{pa^{*}(j)}\right)$ at $x_{j}+1$ and $x_{j}$. We observe that if $x_{j}\neq 0$,

(16)
$$\frac{\left(a_{j1}+x_{j}\right)\cdots \left(a_{jp_{j}}+x_{j}\right)H_{j}\left(\sum_{k\in pa^{\cap }(j)}g_{\text{jk}}\left(x_{k}\right)+\nu_{j}\right)}{\left(b_{j1}+x_{j}\right)\cdots \left(b_{jq_{j}}+x_{j}\right)\left(x_{j}+1\right)}\;=\frac{\left(a_{j1}^{*}+x_{j}\right)\cdots \left(a_{jp_{j}}^{*}+x_{j}\right)H_{j}\left(\sum_{k\in pa^{\cap }(j)}g_{\text{jk}}^{*}\left(x_{k}\right)+\nu_{j}^{*}\right)}{\left(b_{j1}^{*}+x_{j}\right)\cdots \left(b_{jq_{j}}^{*}+x_{j}\right)\left(x_{j}+1\right)},$$

and otherwise,

(17)
$$\frac{a_{j1}\cdots a_{jp_{j}}H_{j}\left(\sum_{k\in pa^{\cap }(j)}g_{\text{jk}}\left(x_{k}\right)+\nu_{j}\right)}{b_{j1}\cdots b_{jq_{j}}\left\{1+\text{exp}{\left(\sum_{k\in pa^{\cap }(j)}f_{\text{jk}}\left(x_{k}\right)+\mu_{j}\right)}_{p_{j}}F_{q_{j}}\left(a_{j};b_{j};H_{j}\left(\sum_{k\in pa^{\cap }(j)}g_{\text{jk}}\left(x_{k}\right)+\nu_{j}\right)\right)\right\}}\;=\frac{a_{j1}^{*}\cdots a_{jp_{j}}^{*}H_{j}\left(\sum_{k\in pa^{\cap }(j)}g_{\text{jk}}^{*}\left(x_{k}\right)+\nu_{j}\right)}{b_{j1}^{*}\cdots b_{jq_{j}}^{*}\left\{1+\text{exp}{\left(\sum_{k\in pa^{\cap }(j)}f_{\text{jk}}^{*}\left(x_{k}\right)+\mu_{j}^{*}\right)}_{p_{j}}F_{q_{j}}\left(a_{j}^{*};b_{j}^{*};H_{j}\left(\sum_{k\in pa^{\cap }(j)}g_{\text{jk}}^{*}\left(x_{k}\right)+\nu_{j}^{*}\right)\right)\right\}}.$$

Note that (16) and (17) hold for all possible values of $x_{k}$ for $k\in pa^{\cap }(j)$. If it is combined with $E\left[f_{\text{jk}}\left(X_{k}\right)\right]=E\left[g_{\text{jk}}\left(X_{k}\right)\right]=0$, our identifiability condition for the nonlinear functions $f_{\text{jk}}$ and $g_{\text{jk}}$, we get $\mu_{j}=\mu_{j}^{*},\nu_{j}=\nu_{j}^{*}$, and $f_{\text{jk}}\left(x_{k}\right)=f_{\text{jk}}^{*}\left(x_{k}\right)$ and $g_{\text{jk}}\left(x_{k}\right)=g_{\text{jk}}^{*}\left(x_{k}\right)$ for all possible values of $x_{k}$ for $k\in pa^{\cap }(j)$. Then, it is clear that $a_{j}$ and $b_{j}$, respectively, are equivalent to a permutation of $a_{j}^{*}$ and $b_{j}^{*}$, which completes the proof.■
